# Inhibition of TBL1 cleavage alleviates doxorubicin-induced cardiomyocytes death by regulating the Wnt/β-catenin signal pathway

**DOI:** 10.1093/cvr/cvae098

**Published:** 2024-05-09

**Authors:** Sun-Ho Lee, Jangho Lee, Jaewon Oh, Jin-Taek Hwang, Hae-Jeung Lee, Hwa Kyung Byun, Hyeong-Jin Kim, David Suh, Ho-Geun Yoon, Sahng Wook Park, Seok-Min Kang, Chulan Kwon, Seung-Hyun Lee, Hyo-Kyoung Choi

**Affiliations:** Department of Biochemistry and Molecular Biology, Graduate School of Medical Science, Brain Korea 21 Project, Yonsei University College of Medicine, Seoul 03722, Republic of Korea; Korea Food Research Institute, Jeollabuk-do 55365, Republic of Korea; Division of Cardiology, Severance Cardiovascular Hospital, Cardiovascular Research Institute, Yonsei University College of Medicine, Seoul 03722, Republic of Korea; Korea Food Research Institute, Jeollabuk-do 55365, Republic of Korea; Department of Food and Nutrition, Gachon University, Gyeonggi-do 13120, Republic of Korea; Department of Radiation Oncology, Yonsei University College of Medicine, Seoul 03722, Republic of Korea; Department of Biochemistry and Molecular Biology, Graduate School of Medical Science, Brain Korea 21 Project, Yonsei University College of Medicine, Seoul 03722, Republic of Korea; Division of Cardiology, Department of Medicine, Johns Hopkins University, Baltimore, MD 21205, USA; Department of Biochemistry and Molecular Biology, Graduate School of Medical Science, Brain Korea 21 Project, Yonsei University College of Medicine, Seoul 03722, Republic of Korea; Institute of Genetic Science, Yonsei University College of Medicine, Seodaemun-gu, Seoul 03722, Republic of Korea; Department of Biochemistry and Molecular Biology, Graduate School of Medical Science, Brain Korea 21 Project, Yonsei University College of Medicine, Seoul 03722, Republic of Korea; Institute of Genetic Science, Yonsei University College of Medicine, Seodaemun-gu, Seoul 03722, Republic of Korea; Division of Cardiology, Severance Cardiovascular Hospital, Cardiovascular Research Institute, Yonsei University College of Medicine, Seoul 03722, Republic of Korea; Division of Cardiology, Department of Medicine, Johns Hopkins University, Baltimore, MD 21205, USA; Department of Biochemistry and Molecular Biology, Graduate School of Medical Science, Brain Korea 21 Project, Yonsei University College of Medicine, Seoul 03722, Republic of Korea; Division of Cardiology, Department of Medicine, Johns Hopkins University, Baltimore, MD 21205, USA; Institute of Genetic Science, Yonsei University College of Medicine, Seodaemun-gu, Seoul 03722, Republic of Korea; Korea Food Research Institute, Jeollabuk-do 55365, Republic of Korea

**Keywords:** Transducin beta-like protein 1, Doxorubicin, Cardiotoxicity, Wnt/β-catenin signal pathway, Human induced pluripotent stem cell derived cardiomyocytes

## Abstract

**Aims:**

Doxorubicin (DOX) is a widely used anthracycline anticancer agent; however, its irreversible effects on the heart can result in DOX-induced cardiotoxicity (DICT) after cancer treatment. Unfortunately, the pathophysiology of DICT has not yet been fully elucidated, and there are no effective strategies for its prevention or treatment. In this investigation, the novel role of transducin beta-like protein 1 (TBL1) in developing and regulating DICT was explored.

**Methods and results:**

We observed a reduction in TBL1 protein expression levels as well as cleavage events in the transplanted cardiac tissues of patients diagnosed with Dilated Cardiomyopathy and DICT. It was revealed that DOX selectively induces TBL1 cleavage at caspase-3 preferred sites—D125, D136, and D215. Interestingly, overexpression of the uncleaved TBL1 mutant (TBL1^uclv^) variant reduced apoptosis, effectively preventing DOX-induced cell death. We confirmed that cleaved TBL1 cannot form a complex with β-catenin. As a result, Wnt reporter activity and Wnt target gene expression collectively indicate a decrease in Wnt/β-catenin signalling, leading to DICT progression. Furthermore, the cleaved TBL1 triggered DOX-induced abnormal electrophysiological features and disrupted calcium homeostasis. However, these effects were improved in TBL1uclv-overexpressing human-induced pluripotent stem cell-derived cardiomyocytes. Finally, in a DICT mouse model, TBL1uclv overexpression inhibited the DICT-induced reduction of cardiac contractility and collagen accumulation, ultimately protecting cardiomyocytes from cell death.

**Conclusion:**

Our findings reveal that the inhibition of TBL1 cleavage not only mitigates apoptosis but also enhances cardiomyocyte function, even in the context of DOX administration. Consequently, this study's results suggest that inhibiting TBL1 cleavage may be a novel strategy to ameliorate DICT.

## Graphical Abstract

**Graphical Abstract:**
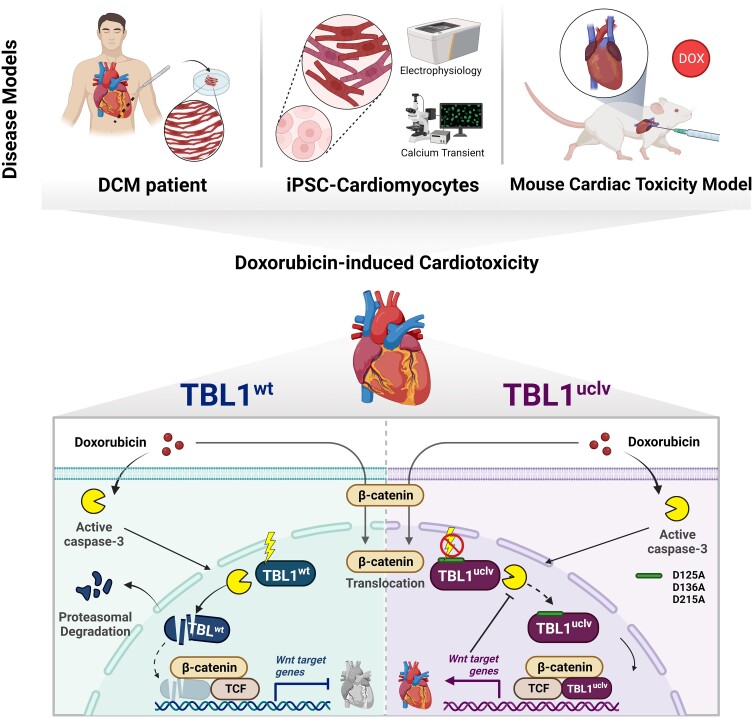

**Time of primary review: 45 days**

## 1. Introduction

Doxorubicin (DOX) is an anthracycline chemotherapeutic agent discovered approximately half a century ago. It is actively used to treat a broad spectrum of malignancies in adults and children due to its high efficacy.^1^ However, as with other anthracyclines, the clinical use of DOX also causes cumulative and dose-dependent cardiotoxicity. The incidence of DOX-induced cardiotoxicity (DICT) is approximately 3–5% at 400 mg/m^2^ and up to 48% at >700 mg/m^2^.^2,3^ After its onset, DICT causes arrhythmias with ventricular de-repolarization disturbances and decreases in left ventricular (LV) function, leading to dilated cardiomyopathy (DCM) and congestive heart failure.^4,5^ DICT has also been shown to occur acutely and chronically.^6^ The mortality rate after a heart failure diagnosis is approximately 50% over a five-year follow-up period.^7^ DICT is consequently of global interest owing to the increasing number of cancer survivors who have received DOX treatment. While DICT is closely associated with a fatal prognosis, we currently lack suitable biomarkers to enable early detection or the development of effective treatment strategies.^8^ Furthermore, the molecular mechanisms underlying DICT still need to be elucidated.

The Wnt/β-catenin signalling pathway is important for many aspects of development and homeostasis, including cell proliferation and migration, apoptosis, and genetic stability.^9^ This pathway is especially important, however, as it has protective effects against chemotherapeutic agents such as DOX in cardiomyocytes and cancer. In an animal model, Wnt inhibitors such as Dickkopf WNT signalling pathway inhibitor 1 (Dkk1) and Protein kinase C-ζ (PKC-ζ) exacerbate DICT.^10,11^ These studies show that adult cardiomyocytes, normal cells, are also subject to the DOX avoidance strategy used in cancer. Recent studies, in particular, have shown that overexpression DEAD-Box helicase 3 X-linked protein ameliorated the DIC response by activating Wnt/β-catenin signalling in cardiomyocytes.^12^ Given that genetic cardiomyopathy is directly related to the incidence of DICT,^13^ it is believed that the Wnt signalling regulator could be an important regulatory factor. These discrepancies further reveal the diversity in heart disease pathogenesis. Overall, the data indicate that Wnt/β-catenin signalling is important in disease pathogenesis and that it appears to broaden the cellular spectrum of DICT.

Transducin beta-like protein 1 (TBL1), which contains F-box and WD-40 domains, was initially identified as a component of the nuclear receptor co-repressor (NCoR)/silencing mediator for retinoid and thyroid hormone receptor (SMRT) co-repressor complex with histone deacetylase 3 (HDAC3).^14,15^ Subsequently, TBL1, as an E3 ubiquitin ligase adaptor, degrades co-repressor complexes through the recruitment of specific ubiquitin/proteasome machinery, leading to the exchange of NCoR/SMRT co-repressors for co-activators upon ligand binding.^16^ Recent efforts to reveal the relationship between TBL1 and Wnt signalling showed that TBL1 and β-catenin recruit each other to the promoter regions of *c-MYC* and *AXIN2*, Wnt target genes, and facilitate transcriptional activation and tumorigenesis.^17^ In addition, the sumoylation of TBL1 uncoupled TBL1 from the NCoR co-repressor complex and conversely interacted with β-catenin, ultimately leading to the activation of Wnt target genes.^18^ In response to agents that cause DNA damage, p53-dependent Siah stabilization contributes to the formation of complexes comprised of Siah-interacting protein, Skp1, and TBL1, affecting the activity of the β-catenin-Tcf/LEF transcription factor.^19^ More recently, TBL1 was found to be required to suppress cardiomyocyte hypertrophy by facilitating interactions between HDAC3 and GATA4.^20^ However, it remains unclear how TBL1 regulates heart function and its signalling.

To address this gap in our knowledge, we suggest a novel function of TBL1 protein as a regulator of Wnt/β-catenin signalling in DICT. We believe these results will help determine whether TBL1 could potentially act as a novel target for ameliorating DICT and aid in developing new clinical applications for DOX.

## 2. Methods

### 2.1 Study samples

LV samples for experimental use were taken from the explanted hearts of patients with DCM who were undergoing cardiac transplantation. The clinical history, haemodynamic analysis, electrocardiogram, and Doppler echocardiogram data for each patient was collected. Patients determined to have LV systolic failure (ejection fraction (EF)<40%) and a dilated non-hypertrophic left ventricle (LV dimension in end-diastole (LVEDD) > 55 mm) using echocardiography were identified as having idiopathic DCM (*n* = 6) and DICT (*n* = 2). Additionally, neither primary valvular nor ischemic heart diseases were present in the selected individuals. According to the functional classification established by the New York Heart Association (NYHA),^21,22^ all patients were receiving medical care in accordance with the guidelines of the European Society of Cardiology. Transmural samples from the apex of the left ventricle were collected and maintained at −80°C until RNA and protein extractions were performed. A comprehensive list of participant characteristics is shown in Supplementary material online, *Table S1*. This study was approved by the Institutional Review Board and ethics committee of the Yonsei University Health System (No. 4-2022-0853). Informed consent was obtained from all patients and the study was performed in accordance with Helsinki declaration. Non-diseased donor hearts were used as control samples (*n* = 15) for immunohistochemistry (IHC), and the tissues were stained with antibodies recognizing TBL1 for IHC analysis. The formalin-fixed and paraffin-embedded tissue microarray comprised 24 samples were obtained from normal cardiac muscle tissues.

### 2.2 Cell culture and cardiomyocyte differentiation

The rat myoblast cell line H9c2 was obtained from the American Type Culture Collection (Manassas, VA, USA) and maintained in Dulbecco’s modified Eagle medium (DMEM; Gibco-RBL, MD, USA) supplemented with 10% fetal bovine serum (Gibco-RBL, MD, USA) and 1% antibiotic–antimycotic (Gibco-RBL) in humidified 5% CO_2_ at 37°C. Normal human cord blood cell-derived iPSC lines (CMC-hiPSC-011), provided by the National Stem Cell Bank of Korea (Korea National Institute of Health, Cheongju, Korea), were originally provided by the Catholic University (Seoul, Korea). These hiPSCs were grown in TeSR-E8 medium (Stemcell Technologies, Vancouver, BC, Canada) on vitronectin-coated plates and passaged every four days using ReLeSR reagent (Stemcell Technologies). Cells were differentiated into cardiomyocytes according to a previously established protocol.^23^ Briefly, cells were seeded on Matrigel-coated six-well plates. They were incubated at approximately 90% confluence in RPMI1640+B27 medium (Gibco-RBL) without insulin and 10 µM CHIR99021 (Tocris, Minneapolis, MN, USA) to activate Wnt signalling and induce mesoderm differentiation. After three days, the media was changed to RPMI1640+B27 without insulin+C59 (Selleckchem, Boston, MA, USA). Then, after 48 h, the medium was changed to RPMI1640+B27 without insulin or B27 with insulin. Cells were then metabolically purified from other differentiated cells by performing glucose deprivation in RPMI1640 without glucose+B27 with insulin as previously described.^24^

### 2.3 Animal experiment

C57BL/6Jms Slc mice (eight-week-old) were obtained from the Japan Sankyo Labo Service Corporation (Shizuoka, Japan). All animal experimental procedures in this study were approved by Yonsei University Health System Institutional Animal Care and Use Committee (IACUC approve No. 2018-0269, 2022-0033); on the basis of the Guide for the Care and Use of Laboratory Animals published by the US National Institutes of Health (NIH Publication No. 85-23, revised 2011, PHS approved Animal Welfare Assurance code F20-00488; Yonsei University Health System). Inhalation anaesthetic was administered in an induction chamber with 5% isoflurane in 100% oxygen at a constant flow rate of 1.0 litres per minute for 5 min. The animals were ventilated with a ventilator (VentElite small rodent ventilator, 55-7040) at 120 respirations per minute with a 2.4 mL stroke volume. Anaesthesia was maintained in 2% isoflurane mixed with 100% oxygen throughout the surgery. Sterile lubricating eye ointment (prednilone) was applied following induction of anaesthesia to prevent corneal drying. The isoflurane ventilation was reduced to zero after completion of the surgery. Once spontaneous breathing was evident, the endotracheal tube was removed. Then, C57BL/6 mice were injected with either AAV9-TBL1^wt^ or AAV9-TBL1^uclv^ via single intramyocardial injections (i.c.). A dosage of 1 × 10^9^ plaque-forming units per mouse was used for adeno-associated virus delivery. To induce cardiotoxicity, DOX (5 mg/kg) was intraperitoneally (i.p.) injected for four weeks (cumulative dose, 20 mg/kg) following the adeno-associated virus injection. The mice used in the *in vivo* experiments were euthanized humanely using CO_2_ gas. Heart tissue was harvested after mice were sacrificed, and tissues were fixed in 4% paraformaldehyde and then embedded in paraffin for serial sectioning.

### 2.4 Multi-electrode array recording and analysis

The iPSC-derived cardiomyocytes were seeded onto a 50 mg/mL fibronectin-coated CytoView MEA 24-well plate (Axion Biosystems, Zurich, Switzerland) at a density of 50,000 cells per well seven days prior to each assay. The activity was recorded before treatment (baseline), just after the DOX treatment (0 h), and 24 h post-treatment with DOX using the Maestro Edge MEA system (Axion Biosystems). DMSO was used as the vehicle control, and an equal volume of vehicle control or DOX was added to the wells. Signals were filtered using a band-pass filter ranging from 0.1 to 2.0 kHz, and the beat detection threshold was 300 µV. Field potentials were analyzed using the platform software, and outputs included beat period (s), spike amplitude (µV), and field potential duration (FPD, ms). Raw FPD measurements were also corrected using Fredericia's rate correction algorithm (FPDcF), where FPDcF = FPD/Beat period^0.33^. All recordings were captured using the standard cardiac settings (AxionBiosystems Maestro AxIS software version 2.1.1.5) at 37°C.

### 2.5 Statistical analysis

To assess data normality, the distribution was evaluated using the Shapiro–Wilk normality test. The obtained results, with a *P*-value ≥ 0.05, confirmed the normality of the data. Subsequently, the data were analyzed using Student's *t*-test or one-way analysis of variance with Tukey's multiple comparison test. Values are expressed as the mean ± SD. Statistical analyses were conducted using Prism 8 (GraphPad Software, La Jolla, CA, USA). Statistical significance was set at *P < 0.05, **P < 0.01, ***P < 0.005, and ****P < 0.0005.

## 3. Results

### 3.1 TBL1 levels are low in the hearts of DCM patients

**Figure 1:**
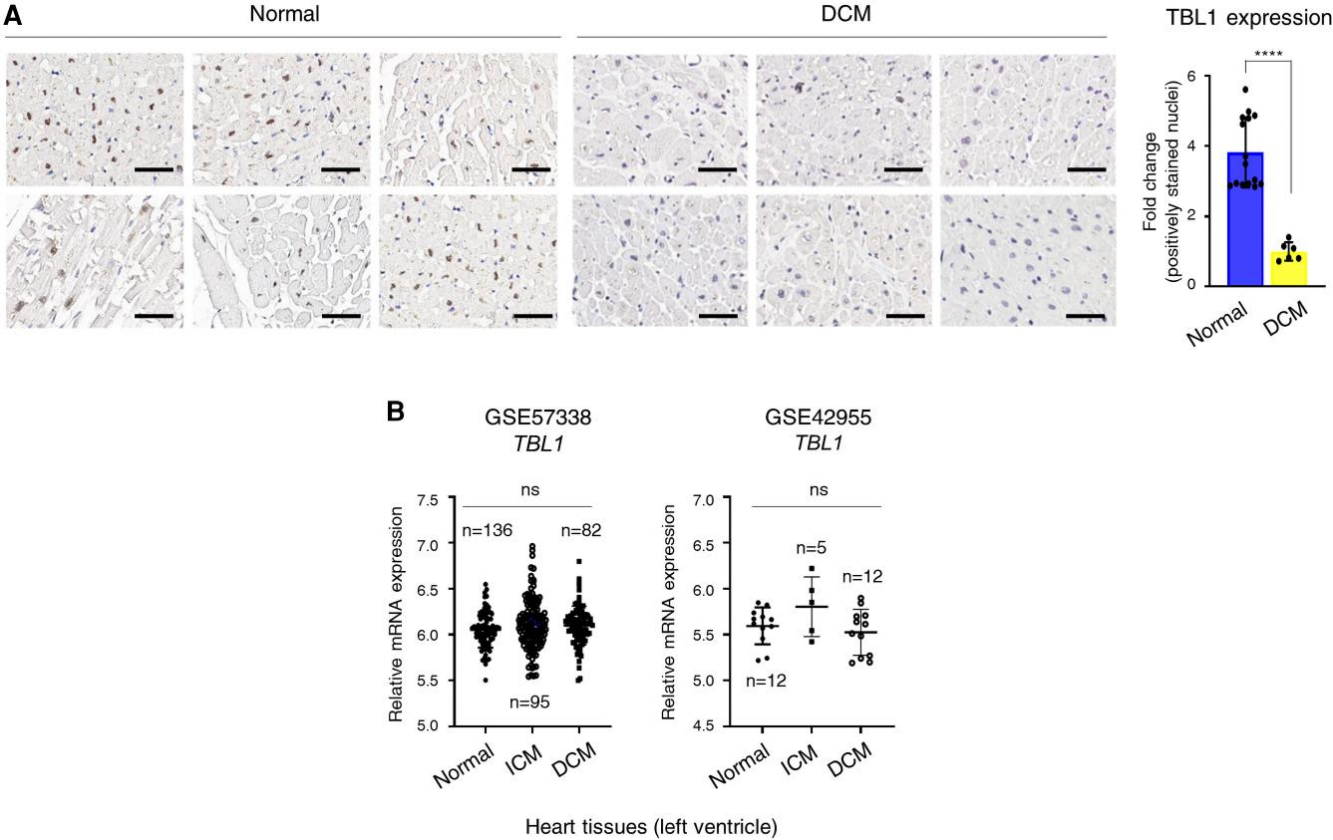
TBL1 expression is low in cardiac tissues from patients with DCM. (*A*) TBL1 expression in cardiac tissues from patients with idiopathic DCM (*n* = 6) compared to normal cardiac tissues (*n* = 15). TBL1 expression is low in the cardiac tissues of patients with DCM. Slides were stained with an antibody against an N-terminal TBL1 epitope using IHC. Representative images are shown. Scale bar = 50 µm (left panel). The DAB (3,3′-diaminobenzidine) staining intensity score was calculated using the IHC profiler plugin in ImageJ software (right panel). ****P < 0.0001 (Student's *t*-test) (*B*) Data from publicly available datasets on TBL1 expression levels in cardiac tissues from patients with DCM and normal cardiac tissues. GSE57338, *n* = 313 (normal *n* = 136, iCMP *n* = 95, iDCMP *n* = 82); GSE42955 *n* = 29 (normal *n* = 5, iCMP *n* = 12, DCMP *n* = 12). Quantification of *TBL1* mRNA levels by microarray (two probes; ILMN_1248994 and ILMN_2624451) in the GSE57337 and by RNA sequencing in GSE42955. n.s., no significance.

To determine whether DCM leads to alterations in the levels of TBL1, its expression was compared between cardiac tissues from DCM patients and normal individuals. The tissues were stained with antibodies recognizing TBL1 for immunohistochemical analysis. TBL1 expression levels were reduced by approximately 75% in the DCM patients (*Figure 1A* and Supplementary material online, *Figure S1*). To check whether this reduction was due to a decrease in *TBL1* transcription in DCM patients, microarray data from the Gene Expression Omnibus (GEO) were analyzed. Among the GEO datasets, the mRNA expression of TBL1 did not change between normal individuals and DCM patients (*Figure 1B*). Taken together, these results indicate that the TBL1 expression level changes in the patients with DCM occurred at a post-translational level.

### 3.2 DOX-induced caspase-3 dependent TBL1 cleavage at D125, D136, and D215, accelerates its proteasomal degradation

**Figure 2:**
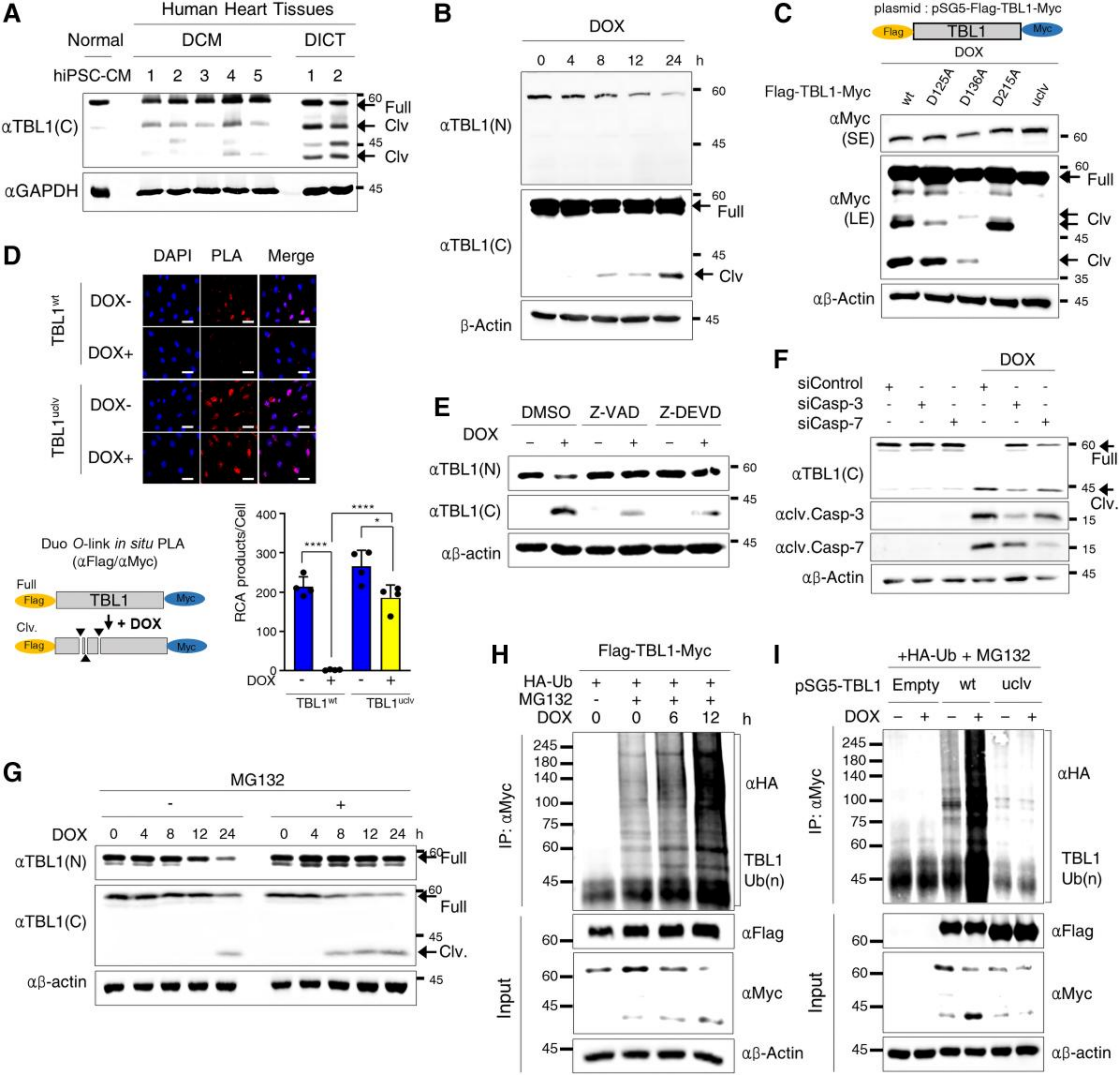
TBL1 is cleaved at D125, D136, and D215 in a caspase-3-dependent manner and consequently destabilized through ubiquitin-dependent degradation following doxorubicin treatment in H9c2 cells. (*A*) TBL1 cleavage in heart tissues sampled from patients with idiopathic DCM and DICT. Proteins were immunoblotted with the indicated antibodies. The arrows indicate cleaved-TBL1. (*B*) Endogenous TBL1 was cleaved in a time-dependent manner following DOX treatment. H9c2 cells were treated with 2 µM DOX, and total proteins were extracted and immunoblotted with the indicated antibodies. SE, short exposure; LE, long exposure. (*C*) TBL1 cleaved at D125, D136, and D215 in response to DOX. Wild-type TBL1 (TBL1^wt^), TBL1^D125A^, TBL1^D136A^, TBL1^D215A^, or mutations at three sites (TBL1^uclv^) were overexpressed in H9c2 cells which were then harvested and lysed to extract the protein. The total protein was used for immunoblot assays. The arrow marked as ‘Clv’ indicate cleaved TBL1 bands. SE, short exposure; LE, long exposure. (*D*) TBL1^uclv^ abrogates its cleavage in response to DOX. H9c2 cells were transiently transfected with a double-tagged TBL1 (Flag-TBL1-Myc) plasmid and treated with DOX. Permeabilized cells were incubated with antibodies against Flag and Myc, and PLA probes added. The cell nuclei were counterstained with DAPI. Dot signals represent RCA products from immuno-RCA reactions detected using fluorescently labelled complementary oligonucleotides. Representative images of four independent experiments are shown. Scale bar = 50 µm (upper panel). The number of RCA products per cell from these images is shown in the bar graph. The values are presented as the mean ± SD from three independent experiments (lower panel). *P < 0.05 and ****P < 0.0001 (Student’s *t*-test). (*E*) TBL1 cleavage was blocked following the treatment of caspase inhibitors. H9c2 cells were exposed to either pan-caspase inhibitor (Z-VAD) or caspase 3/7 specific inhibitor (Z-DEVD) with or without 2 µM DOX for 24 h. The cells were lysed, and protein extracts were used for immunoblot assays with the indicated antibodies. (*F*) In the DOX response, TBL1 is cleaved in a caspase-3-dependent manner. siCaspase-3 or siCaspase-7 was transiently transfected, and subsequently, H9c2 cells were treated with 2 µM DOX for 24 h. Protein extracts from the cells were immunoblotted with the indicated antibodies. (*G*) MG132 treatment induces the accumulation of TBL1 cleavage. Endogenous TBL1 was cleaved in a time-dependent manner following DOX treatment. The cells were treated with 1 µM DOX for the indicated time and 10 µM MG132 for 12 h. Cells were incubated for the indicated time, and whole-cell lysates were immunoblotted using the indicated antibodies. Arrows indicate cleaved TBL1. (*H*) TBL1 ubiquitination increases in response to DOX in a time-dependent manner. H9c2 cells were transfected HA-tagged Ub plasmid and treated with 10 µM MG132 with or without 1 µM DOX for the indicated time. Whole-cell lysates were immunoprecipitated with an anti-TBL1(C) antibody and immunoblotted with the indicated antibodies. (*I*) The TBL^uclv^ mutation abolishes DOX-induced TBL1 ubiquitination. H9c2 cells were transfected with HA-Ub and the indicated Myc-tagged TBL1 plasmids and then treated with 10 µM MG132 with or without 1 µM DOX for 24 h. Whole-cell lysates were immunoprecipitated with anti-Myc antibody and immunoblotted.

The possibility of TBL1 cleavage in patients with idiopathic DCM and DICT was explored (*Figure 2A*). Notably, in patients with both idiopathic DCM and DICT, two distinct bands were observed, which suggested TBL1 cleavage. These bands were positioned beneath the bands corresponding to the full-length TBL1. This phenomenon was more pronounced in the DICT patient group. Normal human induced pluripotent stem cell derived cardiomyocytes (hiPSC-CMs) were used as a control. TBL1 is highly homologous in humans and rats (see Supplementary material online, *Figure S2*); thus, to examine whether DICT induces TBL1 changes, TBL1 was identified after the DOX treatment in H9c2 cells, rat cardiomyocytes, using two separate antibodies against its N- or C-termini. Concordantly, a decrease in endogenous TBL1 expression was observed in both antibodies, but interestingly, the cleaved-TBL1 was detected only when using the C-termini antibody in a time-dependent manner after the DOX treatment (*Figure 2B*). Seven caspase-dependent candidate sites were identified in TBL1 that were found to be critical for DOX-mediated cleavage when using bioinformatics analysis (see Supplementary material online, *Figure S3*). Based on the prediction results, several different constructs were generated to distinguish the TBL1 cleavage region (see Supplementary material online, *Figure S4A*). Each construct was transiently transfected and treated with DOX for 24 h in H9c2 cells. The cleaved TBL1 bands were detected in cells overexpressing Flag-TBL1^1–215aa^ or Flag-TBL1^1–136aa^ and Flag-TBL1^wt^ (see Supplementary material online, *Figure S4B*). To identify the specific cleavage sites, a TBL1 construct double tagged with Flag and Myc at its N- and C-termini was generated (Flag-TBL1-Myc), and it replaced TBL1 Asp-125 (D125), Asp-136 (D136), or Asp-215 (D215) with Ala (A) through site-direct mutagenesis. An additional TBL1 mutant was generated in which all three sites were substituted with A (uncleaved mutant; uclv) (see Supplementary material online, *Figure S5*). Each plasmid was overexpressed in H9c2 cells and then treated with DOX. Among the three cleaved-TBL1 bands, the 49 kD fragment disappeared at TBL1^D125A^, the 47.7 kD fragment at TBL1^D136A^, and the 40 kD fragment at TBL1^D215A^. However, cleaved-TBL1 bands were not observed in TBL1^uclv^ (*Figure 2C*). To verify the importance of these three sites in TBL1 for its cleavage, a proximity ligation assay (PLA) was performed using a double Flag-TBL1-Myc construct for the *in situ* visualization and quantification of TBL1 cleavage (see Supplementary material online, *Figure S6*). DOX efficiently induced the cleavage of TBL1^wt^, which failed to produce rolling circle amplification (RCA). However, TBL1^uclv^ successfully formed RCA in DOX-exposed H9c2 cells (*Figure 2D*). To identify whether caspase was involved in TBL1 cleavage following the DOX treatment, caspase assays were performed using an *in vitro* translation system (see Supplementary material online, *Figure S9*). Caspase-3 and -7 predominately caused TBL1 cleavage. To confirm this, a caspase-3/-7 specific inhibitor, Z-DEVD, was used to treat H9c2 cells. Concordantly, the DOX-induced TBL1 cleavage was abrogated following the Z-DEVD treatment in combination with DOX. The treatment with Z-VAD, a pan-caspase inhibitor, exhibited similar results (*Figure 2E*). However, the cleavage of TBL1 following DOX treatment was independent of calpain-1 and calpain-2 (see Supplementary material online, *Figure S7*). A small interfering RNA was used to assess if DOX-induced TBL1 cleavage had specificity to caspase-3 or -7. Notably, following the caspase-3 knockdown, DOX-induced TBL1 cleavage was considerably reduced (*Figure 2F* and Supplementary material online, *Figure S8*). Furthermore, in caspase-3 assays using *in vitro* translation systems, the expected cleaved bands were detected in TBL1wt and TBL1 mutants; however, cleavage was not observed in TBL1^uclv^ (see Supplementary material online, *Figure S10*). These results collectively indicate that DOX induces TBL1 cleavage at D125, D136, and D215 in a caspase-3-dependent manner.

DOX also induces extensive ubiquitination associated with a decrease in protein translation.^25^ Given that the DOX treatment results in TBL1 cleavage and a decrease in its levels, it was further investigated if the degradation was due to its reduction. To do this, TBL1 levels were analyzed after the DOX treatment with or without MG132, a proteasomal inhibitor. The results showed that 8 h after the DOX treatment, approximately 20% of the TBL1 was detected in its cleaved form in the presence of MG132. Then, 24 h after the DOX treatment, over 40% of the full-length TBL1 was cleaved (*Figure 2G*). To complement this, an ubiquitination assay was conducted on H9c2 cells. TBL1 was ubiquitinated following the DOX treatment in a time-dependent manner (*Figure 2H*). To confirm whether ubiquitination of TBL1 was associated with its cleavage, we overexpressed either TBL1^wt^ or TBL1^uclv^ and performed ubiquitination assays. DOX-induced ubiquitination of TBL1^wt^ was substantially increased, whereas TBL1^uclv^ was not ubiquitinated (*Figure 2I*). These results indicate that DOX-induced TBL1 cleavage leads to the ubiquitin-dependent degradation of TBL1.

### 3.3 DOX-induced TBL1 cleavage accelerates apoptotic cell death in H9c2 cells

**Figure 3:**
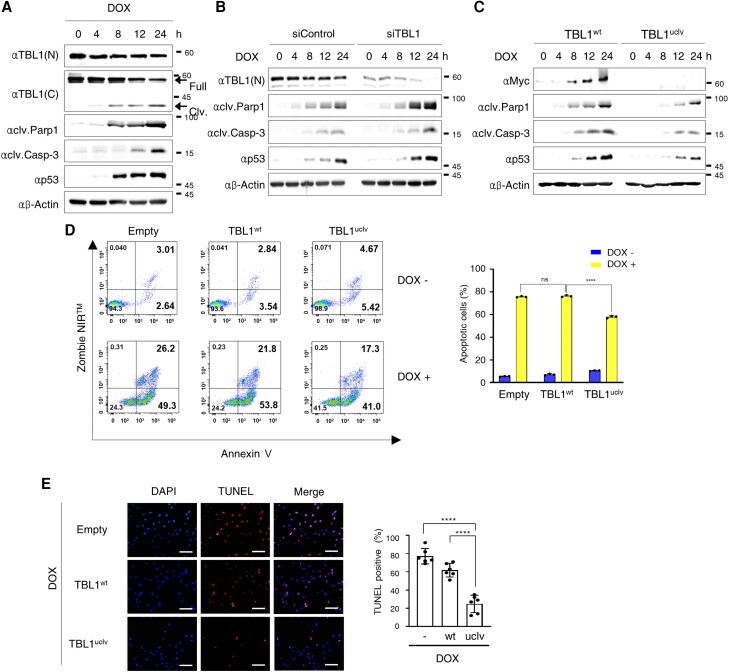
Depletion of TBL1 abrogates the DOX-induced TBL1 cleavage and p53-dependent apoptotic cell death. (*A*) Endogenous TBL1 was cleaved in a time-dependent manner following DOX treatment. The cells were treated with 2 µM DOX for the indicated time. Protein from the H9c2 cells was extracted and immunoblotted with the indicated antibodies. (*B*) Depletion of TBL1 abrogates the DOX-induced TBL1 cleavage and p53-dependent apoptotic cell death. DOX-induced TBL1 cleavage regulated the expression of apoptotic proteins. H9c2 cells were treated with 2 µM DOX for the indicated times. Cells were harvested, and total protein extracted. Whole-cell lysates were immunoblotted with the indicated antibodies. (*C*) TBL1 Knockdown increased the expression of apoptotic proteins in response to the DOX treatment. Either siControl (siCONT) or siTBL1 was transiently transfected in H9c2 cells and treated with 2 µM DOX for the indicated times. Whole-cell lysates were used for immunoblotting assays with the indicated antibodies. (*D*) Abrogation of TBL1 cleavage inhibits apoptotic cell death of rat cardiomyocytes in response to DOX. Either TBL1^wt^ or TBL1uclv construct was transfected into H9c2 cells, and the cells were exposed to 2 µM DOX for 24 h. Annexin V-positive cells were assessed by flow cytometry. A representative image of three independent experiments is shown. (*E*) Representative image of TUNEL-positive apoptotic cells vs. DAPI. Total cells positive for Empty, wild-type TBL1, and uncleaved-TBL mutant overexpression were detected by fluorescent microscopy. Cells were exposed to 2 µM DOX for 24 h. Representative images of three independent experiments are demonstrated (right panel). Scale bar = 20 µm. The histogram depicts the quantification of apoptosis (in percentage; the total number of TUNEL-positive cells vs. DAPI-positive cells) for TBL1^wt^ and TBL1^uclv^. The values are presented as the mean ± SD from three independent experiments. n.s., no significance. ****P < 0.0001 (Student's *t*-test).

To determine if TBL1 is involved in DOX-induced apoptotic cell death in cardiomyocytes, the expression of the predominant factors mediating apoptotic cell death was investigated. Cleaved-PARP1, cleaved–caspase-3, and p53 were increased following DOX-induced TBL1 cleavage (*Figure 3A*). To confirm TBL1 involvement in this phenomenon, the TBL1 was depleted using siRNAs, and the pro-apoptotic factor levels were monitored. The expression of these factors was more highly upregulated in TBL1 knocked-down cells than in control cells (*Figure 3B*). Notably, in response to the DOX treatment, TBL1^uclv^-overexpressing cardiomyocytes showed lower levels of apoptotic factors than in TBL1^wt^-overexpressing cells (*Figure 3C*). Consistently, TBL1^uclv^ overexpression resulted in significantly less apoptotic cell death than TBL1^wt^ overexpression in the presence of DOX (*Figure 3D*). This result was further confirmed by the TUNEL staining (*Figure 3E*). Collectively, these findings suggest that DOX-induced TBL1 cleavage increases the expression of pro-apoptotic factors, resulting in cardiomyocyte death.

### 3.4 DOX-induced TBL1 cleavage blocks the Wnt/β-catenin signal pathway in H9c2 cells

**Figure 4:**
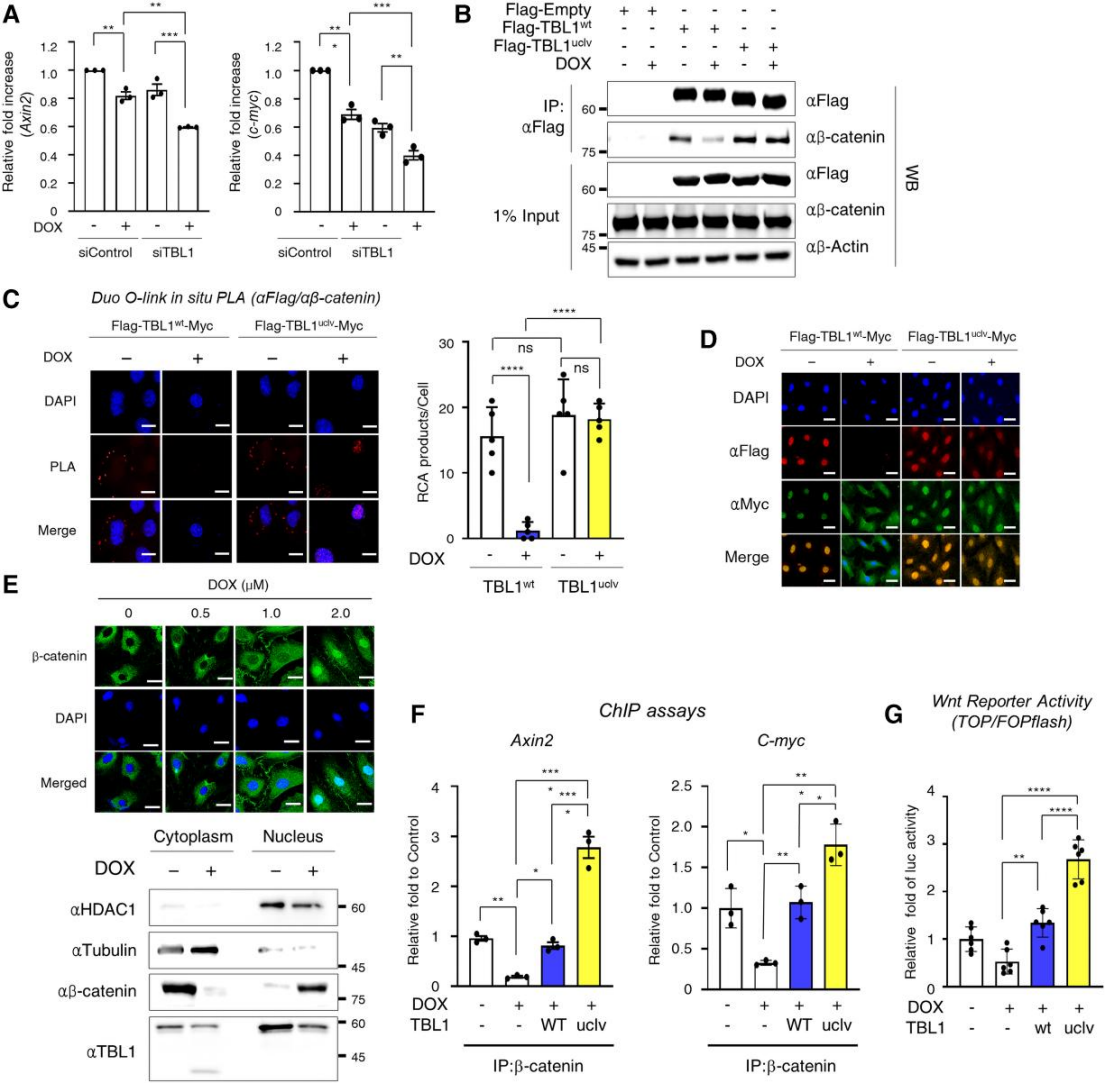
DOX-induced TBL1 cleavage abrogates Wnt/β-catenin signal pathway activation. (*A*) *TBL1* knockdown decreased mRNA expression of the Wnt target genes. H9c2 cells were transfected with either siControl or siTBL1 and exposed to 1 µM DOX for 24 h. cDNA was synthesized from mRNA, and the levels of *Axin2* and *c-Myc* were analyzed using real-time PCR. (*B*) Exogenous overexpressed TBL1^wt^ was dissociated from β-catenin following the DOX treatment. The indicated plasmid was transfected with or without 1 µM DOX for 24 h in H9c2 cells. Whole-cell lysates were immunoprecipitated with a β-catenin antibody and sequentially immunoblotted with the indicated antibodies. (*C*) *In situ* PLA assay demonstrating the dissociation of cleaved TBL1^wt^ from β-catenin in response to DOX. Flag-TBL1^wt^ or -TBL1^uclv^ plasmid was overexpressed with or without 2 µM DOX for 24 h in H9c2 cells. Permeabilized cells were incubated with antibodies against Flag and β-catenin, and the PLA probes were added. The cell nuclei were counterstained with DAPI. Positive signals were analyzed using confocal microscopy. RCA dots indicate the TBL1-β-catenin complex. Representative images of four independent experiments are demonstrated. Scale bar = 50 µm (left panel). The numbers of RCA products per cell from those images are shown in the bar graph (right panel). (*D*) DOX-induced TBL1 cleavage mediates its abnormal localization. The cleaved TBL1^wt^ was relocalized into the cytosol in response to the DOX treatment. Flag-TBL1^wt^-Myc or Flag-TBL1^uclv^-Myc plasmid was transfected into H9c2 cells with or without 1 µM DOX for 24 h. Immunofluorescence analysis was performed as described in the Materials and Methods section. Representative images of three independent experiments are shown. Scale bar = 50 µm. (*E*) DOX induces β-catenin nuclear translocation. Cells were treated with 2 µM DOX for the indicated time. H9c2 cells were analyzed using immunofluorescence staining. Representative images of four independent experiments are demonstrated. Scale bar = 50 µm. H9c2 cells with or without 2 µM DOX for 24 h were fractionized into the cytosol and nucleus and used for western blot assays (lower panel). (*F*) DOX-induced TBL1 cleavage abrogates its occupancy in the promoter region of Wnt target genes. H9c2 cells were transiently transfected with either the TBL1^wt^ or TBL1^uclv^ plasmid with or without 1 µM DOX for 24 h. Chromatin immunoprecipitation assays were performed with the indicated antibodies. Precipitated samples were analyzed using real-time PCR. (*G*) Overexpression of TBL1^uclv^ enhances β-catenin-mediated transcription activity under DOX-exposed conditions. H9c2 cells were co-transfected with TOP/FOPFLASH reporter and the indicated TBL1 plasmid for 24 h. Whole-cell lysates were used for luciferase assays. The results are represented as the mean ± SD from the three independent experiments. n.s., no significance. *P < 0.05, **P < 0.01, ***P < 0.001, and ****P < 0.0001 (Student’s *t*-test).

Following treatment with the Wnt agonists LiCl and CHIR99021, DOX-induced apoptotic cell death was remarkably decreased (see Supplementary material online, *Figure S11*). Based on these results, it was hypothesized that DOX-induced TBL1 cleavage would inhibit the Wnt/β-catenin signalling axis. The major factors involved in the pathway following the TBL1 knockdown were monitored to test this. The depletion of TBL1 significantly reduced the levels of Wnt target genes *Axin2* and *c-myc* in response to the DOX treatment (*Figure 4A*). The IP and PLA analyses showed that the TBL1^uclv^-β-catenin complex was not affected by the DOX treatment (*Figure 4B* and *C*). It was assumed that these binding differences would be seen because the cellular localization of the two proteins, TBL1 and β-catenin, respectively, changed during the DOX treatment. TBL1^wt^ was translocated to the cytosol, but TBL1^uclv^ was still localized in the nucleus in response to the DOX. (*Figure 4D*). Immunostaining was then conducted to determine whether the DOX treatment affects the nuclear translocation of β-catenin. Notably, the DOX treatment induced the nuclear localization of β-catenin in a dose-dependent manner (*Figure 4E*, upper panel), and this was further confirmed by cell fractionization (*Figure 4E*, lower panel). TBL1 has been shown to be recruited to the TCF binding sites alongside β-catenin to activate Wnt targets, such as *Axin2* and *c-Myc*.^17,18^ Consequently, we investigated whether β-catenin occupancy in the loci of Wnt target genes, specifically *Axin2* and *c-Myc*, was affected by TBL1 cleavage after DOX exposure. The chromatin immunoprecipitation (ChIP) assay revealed that β-catenin occupancy becomes significantly higher in TBL1^uclv^-overexpressing cells than in TBL1^wt^-overexpressing cells in the presence of DOX (*Figure 4F*). Concordantly, uncleaved-TBL1 expression significantly increased *Wnt* reporter activity even in the presence of DOX (*Figure 4G*). Furthermore, TBL1 mutants, which were truncated at the cleavage site, failed to increase in both Wnt reporter activity and *Axin2* mRNA expression (see Supplementary material online, *Figure S12*). These results suggest that TBL1 cleavage decreases Wnt/β-catenin activity through its dissociation from β-catenin and that the attenuation leads to apoptotic cell death by enhancing the sensitivity to DOX.

### 3.5 DOX-induced TBL1 cleavage disrupts spontaneous electrophysiological responses and Ca^2+^ homeostasis in hiPSC-CMs

**Figure 5:**
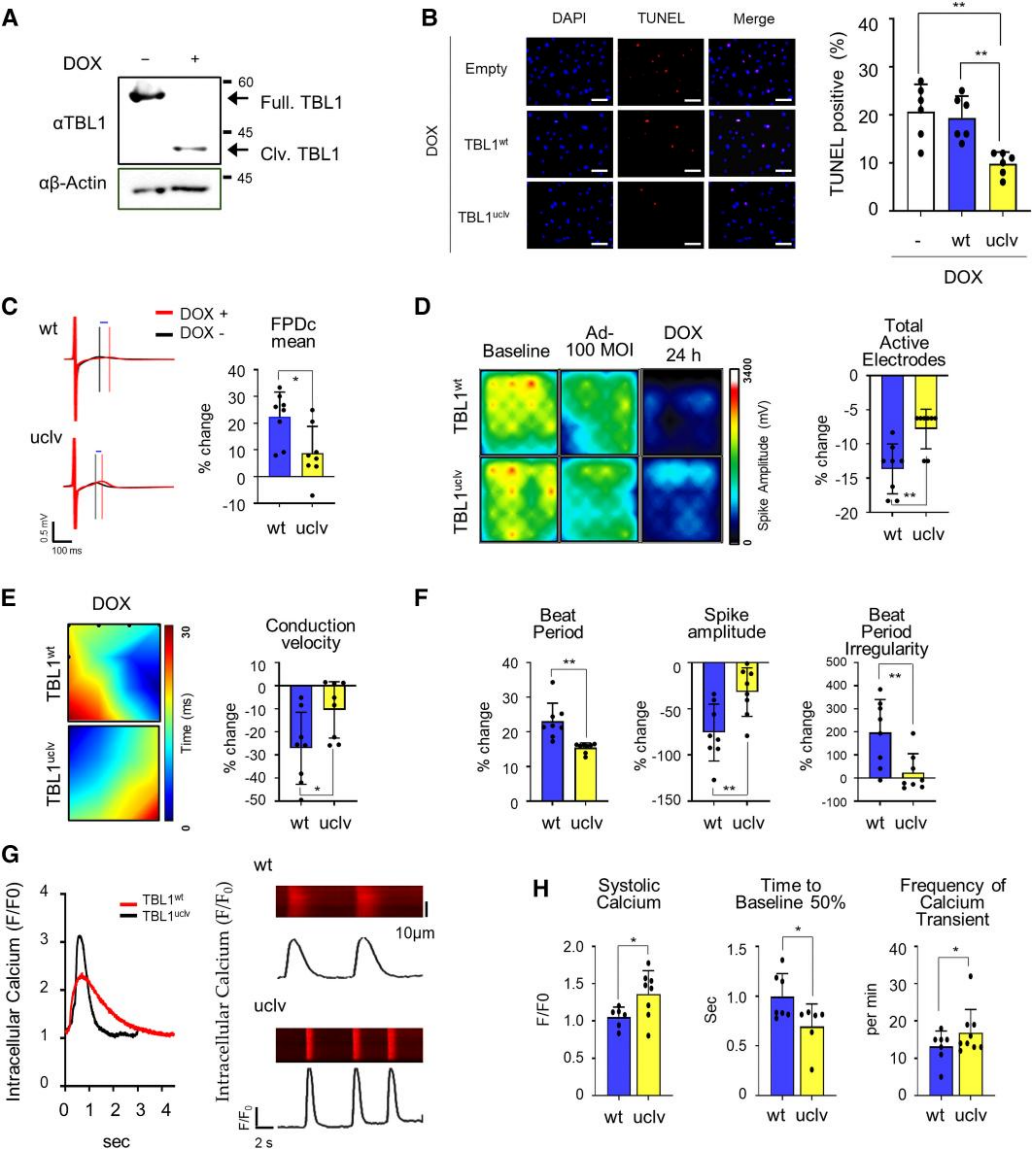
Abrogation of TBL1 cleavage reverses DOX-induced abnormal physiological changes in human-induced pluripotent stem cell-derived cardiomyocytes (hiPSC-CMs). (*A*) iPSC-CMs were cleaved following DOX treatment. iPSC-CMs were treated with 2 µM DOX, and total proteins were extracted and immunoblotted with the indicated antibodies. (*B*) Representative image of TUNEL-positive apoptotic cells vs. DAPI. Ad-Empty, -TBL1^wt^, or Ad-TBL1^uclv^ inoculated into iPSC-CMs and TUNEL-positive cells were detected using fluorescent microscopy. Cells were exposed to 2 µM DOX for 24 h. Representative images of three independent experiments are demonstrated (right panel). Scale bar = 20 µm. The histogram depicts the quantification of apoptosis (in percentage; the total number of TUNEL-positive cells vs. DAPI-positive cells) for TBL1^wt^ and TBL1^uclv^. (*C*) Overexpression of Ad-TBL1^uclv^ in hiPSC-CMs improves field potential in response to DOX. Field potential traces data recorded with the MEA exhibiting mean spontaneous beating trace. Representative trace images recorded with the MEA exhibiting a field potential trace in hiPSC-CMs with the overexpression of Ad-TBL1^wt^ and Ad-TBL1^uclv^ with or without DOX. The graph indicates beat period and spike amplitude. (*D*) Overexpression of Ad-TBL1^uclv^ in hiPSC-CMs improves decreased spike amplitude in response to DOX. An active map through the total active lid count is shown. The degree of the waveform refers to the highs and lows of the spike amplitudes for iPSC-CMs, respectively. Representative images are presented after three independent experiments. (*E*) Overexpression of Ad-TBL1^uclv^ in hiPSC-CMs improves decreased conduction velocity in response to DOX. Conduction plot showing propagation delay of hiPSC-CMs with Ad-Flag-TBL1^wt^ or Ad-Flag-TBL1^uclv^, the two points that appear to be the starting points of the waveform represent the beginning and end of the conduction. Representative images are demonstrated after three independent experiments (left panel). The data with percent changes compared to that of the baseline were measured using MEA (right panel). (*F*) Overexpression of TBL1^uclv^ reversed the disrupted electrophysiological activity in DOX-exposed iPSC-CMs. Ad-TBL1^wt^ or -TBL1^uclv^ was used to infect hiPSC-CMs and exposed to 1 µM DOX. The changes in the beat period, spike amplitude, and beat period irregularity with percent changes compared to that in the baseline were measured using an MEA assay. (*G* and *H*) Overexpression of TBL1^uclv^ attenuates calcium decay in DOX-exposed iPSC-CMs. Representative tracing of spontaneous rhythmic calcium transience in hiPSC-CMs injected with Ad-TBL1^wt^ and -TBL1^uclv^. A typical line scan (X-T mode) image of spontaneous calcium transients was obtained. The systolic calcium (F/F_0_), time to baseline 50% (s), calcium amplitude (F/F_0_), Tau (s), and frequency of calcium transient (per min) were measured. F/F_0_, fluorescence (*F*) normalized to baseline fluorescence (F_0_); s, seconds. The data with percent changes compared to that in the baseline were measured using MEA. The values are the mean ± SD from three independent experiments. * *P* < 0.05 and ** *P* < 0.01 (Student’s *t*-test).

To determine whether DOX-induced TBL1 cleavage affects human cardiomyocyte function, hiPSC-CMs were utilized. The hiPSCs (see Supplementary material online, *Figure S13*) were cultured in defined media and differentiated into cardiomyocytes (see Supplementary material online, *Figure S14A*). For the molecular and structural characterization of the hiPSC-CMs, they were stained using antibodies against TNNT2, NKX2.5, α-actin, titin, and MLC2V (see Supplementary material online, *Figure S14B*–*D*). First, we confirmed that the phenomenon of TBL1 cleavage was also observed in hiPSC-CMs in response to DOX, which was in agreement with our previous findings (*Figure 5A*). The functional analysis of the hiPSC-CMs was performed on a multi-electrode array (MEA) system, which provides non-invasive data recordings. The hiPSC-CMs were cultured with or without the inoculation of adenoviruses (Ad-) harbouring either TBL1^wt^ or TBL1^uclv^ (see Supplementary material online, *Figure S15*) on MEA plates. Data were acquired on the same plate after 9 and 10 days (see Supplementary material online, *Figure S16*). Similarly, hiPSC-CMs expressing TBL1^uclv^ had a lower TUNEL-positive rate than cells expressing TBL1^wt^ (*Figure 5B*).

The MEA-based functionalities of the hiPSC-CMs expressing TBL1 were then investigated. FPD record data showed clearly visible R/Q and T peaks with high signal to baseline ratios in cells expressing TBL1^wt^ or TBL1^uclv^ with/without DOX. QT interval prolongation was, however, lower with TBL1^uclv^ than with TBL1^wt^ under the DOX treatment (*Figure 5C*). With the DOX treatment, TBL1^wt^-expressing cells had a lower spike amplitude, fewer active electrodes, and slower conduction velocity than TBL1^uclv^-expressing cells (*Figure 5D* and *E*, respectively). Beat period and beat period irregularity values were also improved in TBL1^uclv^-expressing cells (*Figure 5F*). To assess calcium cycling, hiPSC-CMs were replated onto Matrigel-coated glass coverslips, and their intracellular calcium transients were measured using Rhod-2, AM (*Figure 5G*). Notably, the decrease in systolic calcium, frequency of the calcium transients, and delated decay of diastolic calcium transients were significantly improved in TBL1^uclv^-expressing hiPSC-CMs (*Figure 5H*). These results suggest that inhibiting TBL1 cleavage alleviates some of the abnormal electrophysiological changes occurring in DICT.

### 3.6 Inhibiting TBL1 cleavage improves cardiac function and ameliorates cardiac fibrosis in mice

**Figure 6:**
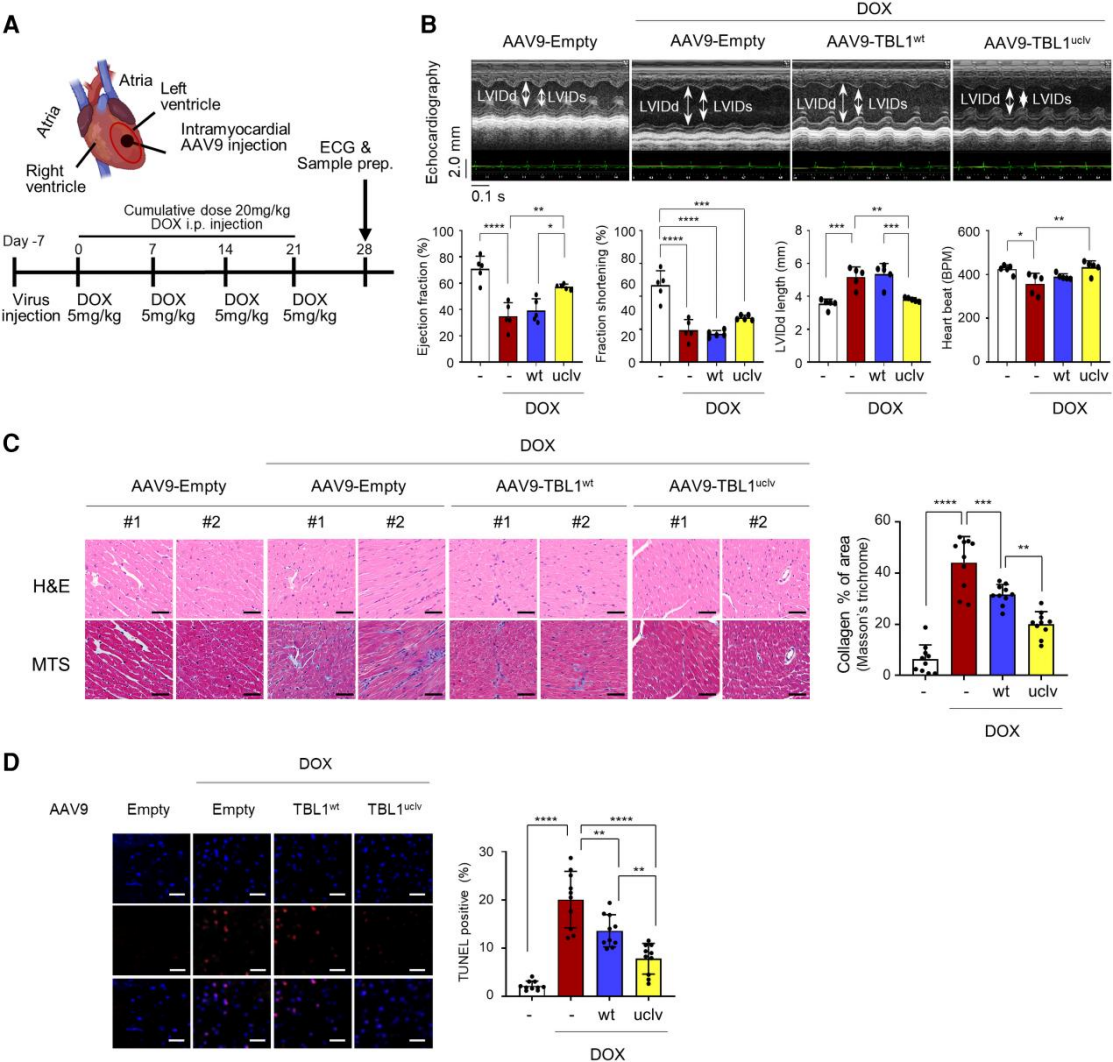
Blocking TBL1 cleavage protects mouse cardiac function by ameliorating genotoxic damage and fibrosis in cardiomyocytes. (*A*) Mouse experimental protocol. The indicated viral particles were injected into the mouse cardiac using a sophisticated closed-chest echocardiography-guided intramyocardial (i.c.) injection method. After viral injection, 5 mg/kg of DOX was intraperitoneally (i.p.) injected four times (cumulative dose, 20 mg/kg). (*B*) Overexpression of AAV9-TBL1^uclv^ protects mouse cardiac function against DICT. After a week from intramyocardial (i.c.) injection of the indicated viral particle and sequential intraperitoneal (i.p.) injection of DOX, mouse cardiac function was observed with echocardiography. Representative M-mode echocardiogram images were demonstrated. LVIDd and LVIDs are marked with a double arrow (upper panel). EF%, FS%, and LVIDd length were measured. The values are demonstrated as the mean ± SE from three independent experiments. *P < 0.05 and **P < 0.01 (Student’s *t*-test; *n* = 5). (*C*) Overexpression of TBL1^uclv^ inhibits DOX-induced cardiac fibrosis in mice. After echocardiography, mice were sacrificed, and their cardiac tissues dissected. The extent of fibrosis was measured using haematoxylin and eosin (H&E) or Masson's trichrome staining (MTS). Two representative images of H&E or MTS staining are demonstrated (left panel). Scale bar = 50 µm. The percent of the collagen-stained area was calculated IHC profiler plugin in ImageJ software (right panel). The values are the mean ± SE from three independent experiments (*n* = 5). **P < 0.01, and ****P < 0.0001 (Student’s *t*-test). (*D*) Inhibition of TBL1 cleavage suppresses mouse cardiomyocyte death. After echocardiography, mice were sacrificed, and their cardiac tissues dissected. DNA damage in the paraffin-embedded cardiac tissues caused by DICT was determined using TUNEL assays. The values are represented as the mean ± SE from three independent experiments. *P < 0.05 and ****P < 0.0001 (Student’s *t*-test). Scale bar = 50 µm.

A DICT mouse model was utilized to validate the *in vitro* findings *in vivo*. To do this, 5 mg/kg of DOX was intraperitoneally injected four times at a cumulative dose of 20 mg/kg after an intramyocardial injection of adeno-associated virus 9 (AAV9) bearing either wild-type or uncleaved-TBL1 (*Figure 6A* and Supplementary material online, *Figure S17*). In AAV9-TBL1^wt^-injected mice, the DOX treatment significantly reduced cardiac contractility as determined by fractional shortening (FS%) and the EF%. This effect was significantly attenuated in TBL1^uclv^-expressing mice; the LV internal dimensions at diastole (LVIDd) were also shorter than in TBL1^wt^-expressing mice and heart rate was also improved (*Figure 6B*). Given that DOX treatment induces cardiac fibrosis through collagen accumulation,^26^ cardiac fibrosis was analyzed using Masson's trichrome staining (MTS). Notably, collagen accumulation was significantly lower in mice expressing TBL1^uclv^ (*Figure 6C*). Likewise, the number of TUNEL-positive cardiomyocytes was significantly decreased in mice expressing TBL1^uclv^ (*Figure 6D*). Together, the data suggest that the inhibition of TBL1 cleavage alleviates DICT *in vivo*.

## 4. Discussion

**Figure 7:**
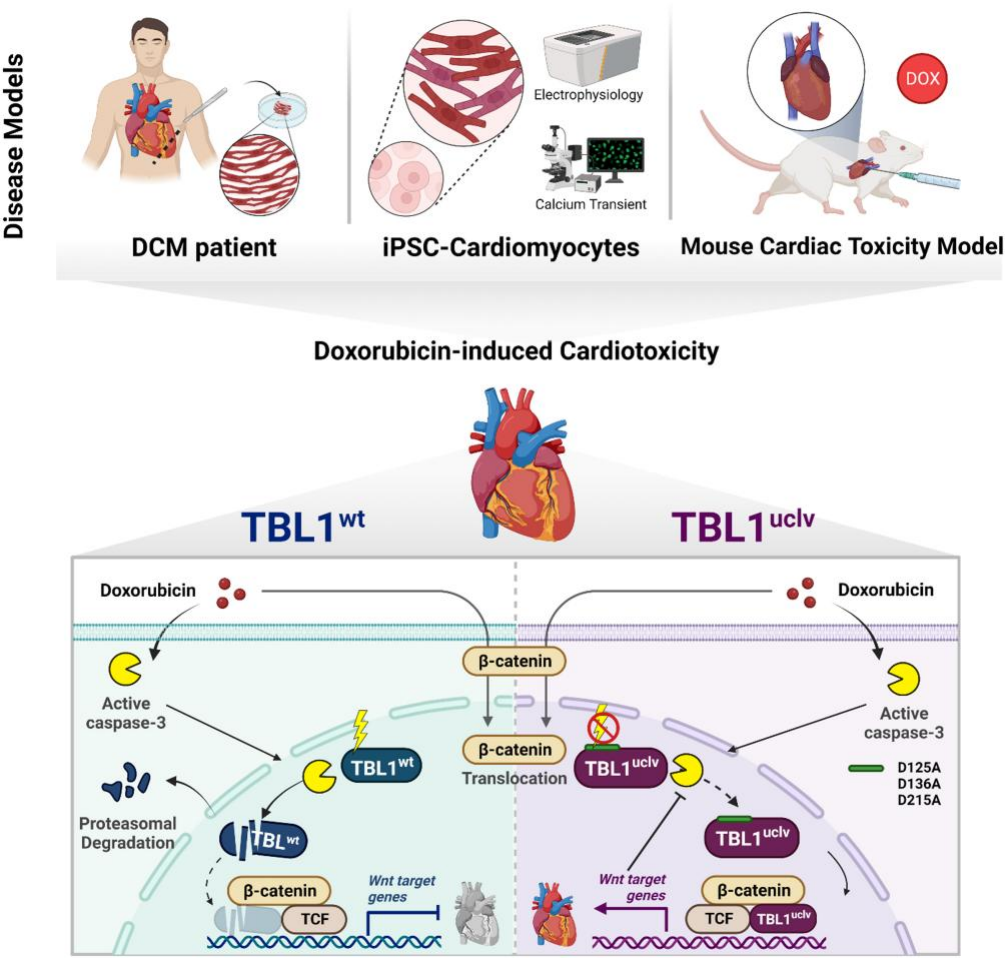
Schematic representation of the major findings from this study. Briefly, DOX-induced TBL1 cleavage at D125, D136, and D215 causes its ubiquitin-dependent degradation. Owing to TBL1 destabilization, it fails to form a complex with β-catenin in the nucleus and blocks β-catenin from occupying the *Axin2* and *c-myc* promoters. Consequently, Wnt activity is decreased, and the death of cardiomyocytes is induced in response to DOX. Therefore, inhibition of TBL1 cleavage protects cardiomyocytes from DICT by controlling the series of signalling mechanisms and consequently maintains normal cardiac function. This image was created with BioRender.com.

Anthracyclines are crucial to many chemotherapy regimens but are also linked to an elevated risk of heart failure. In this regard, it is necessary to elucidate their molecular mechanisms and identify effective preventive and therapeutic targets of DICT. In this study, we have demonstrated that inhibiting TBL1 cleavage reduced the DOX-induced death of the cardiomyocytes. More importantly, TBL1^wt^ failed to activate Wnt signalling despite β-catenin nuclear translocation following DOX administration, but TBL1^uclv^ efficiently activated Wnt signalling, highlighting the importance of TBL1 cleavage and its functions during cardiomyocyte apoptosis. Furthermore, the biological and functional significance of TBL1 cleavage in DICT was elucidated, and the molecular mechanisms were explored via various experiments, including *in vitro*, *in vivo,* and *ex vivo* studies (*Figure 7*).

Evidence has shown beyond a doubt that a protein's cleavage causes destabilization. The cleavage of the gene associated with retinoid interferon-induced mortality (*Grim*) at D136 was found to enhance the stability of *Grim* by removing the Lys residue for ubiquitin conjugation.^27^ While caspase-3-dependent HDAC3 cleavage at D391 was found to ultimately lead to its degradation in etoposide-induced genotoxic stress.^28^ Several post-translational modifications (PTM) of TBL1 including phosphorylation^29^ and sumoylation^18^ have already been reported; however, in this investigation, we have demonstrated its cleavage in DICT for the first time. There is a large amount of evidence showing that protein cleavage, a PTM, induces protein degradation.^30,31^ Considering the TBL1 mRNA expression in the cardiac tissues, the low expression of TBL1 in patients with DCM may be due to cleavage-triggered degradation. In response to DOX, we observed that cleavage occurred at D125, D136, and D215 of the TBL1 in a caspase-3-dependent manner. The cleavage of TBL1 induced its ubiquitin-dependent degradation.

However, the overexpression of TBL1^uclv^ inhibited ubiquitin-dependent proteasomal degradation of TBL1 and facilitated more effective stabilization than that of TBL1^wt^ following DOX exposure. The lis-homology (LisH) domain in the N-terminal plays a crucial role in the maintenance of protein stability^32^ and is located within the 55–87 amino acid range of TBL1, which is important for its half-life.^33^ Based on our results, TBL1 cleavage induced the elimination of the LisH domain through ubiquitin-dependent proteasomal degradation, which is likely how the half-life is shortened. Furthermore, unlike exogenous TBL1, the absence of some cleavage bands of endogenous TBL1 in western blot analysis is probably related to the above reasons.

The question of how TBL1^uclv^, which has a stable status despite the DOX-exposed environment, affects cardiomyocytes was then addressed. To answer this question, the alterations in the key factors^34^ regulating the death of cardiomyocytes in response to DOX were observed. The data showed a time-dependent increase in p53 and pro-apoptotic proteins with DOX-induced TBL1 cleavage. Interestingly, under DOX-exposed conditions, the expression of pro-apoptotic proteins, including p53 and the apoptosis rate in TBL1^uclv^-overexpressing H9c2 cells was lower when compared with those in TBL1^wt^-overexpressing cells. These results indicate that intact TBL1, even in DICT, plays a protective role in cardiomyocytes by suppressing p53. The next aim was to identify which molecular mechanisms were involved in cardiomyocyte death in DICT. The inhibition of Wnt/β-catenin signalling promotes DOX-triggered apoptosis.^35^ DOX induces cardiotoxicity by inhibiting the Wnt/β-catenin signal pathway.^11,36^ A high level of cross-talk between p53 and Wnt signalling has been revealed.^37^ Specifically, activation of p53, a key regulator of apoptosis signalling, leads to the downregulation of the Wnt/β-catenin signalling pathway.^38^ Some studies indicate that the loss of p53 results in the activation of the Wnt/β-catenin signalling pathway.^39,40^ At this time, the regulation of the pathway is determined by the occupancy of p53 at the *Wnt* promoter. Previous findings indicate that the high occupancy of p53 at the site induces the inhibition of the Wnt/β-catenin signal pathway. Therefore, it is possible that TBL1^uclv^-triggered the reduction of p53 and that this may cause inactivation in the pathway following the DOX treatment. One of the most important findings was that TBL1 and β-catenin were recruited to the Wnt target genes, *Axin 2* and *c-myc*, leading to Wnt signalling activation.^17,18^ These results indicate that TBL1 can also directly regulate the Wnt/β-catenin signal pathway. However, studies on TBL1-mediated regulation of the Wnt/β-catenin signal pathway under cellular damage have not yet been conducted in sufficient detail. Our results show that TBL1^uclv^ firmly formed a complex with β-catenin in response to DOX.

Furthermore, ChIP assay analyses and reporter gene assays showed that TBL1^uclv^ dramatically induced the recruitment of β-catenin to the promoter region of *Wnt* target genes, *Axin2* and *c-myc*, and suppressed the decrease in DOX-induced *Wnt* reporter activity. Based on the findings from the immunofluorescence analyses, it is believed that the normal localization of TBL1^unclv,^ even under the DOX-exposed conditions, plays a decisive role in the above results. TBL1 is predominantly localized in the nucleus.^14,15^ Leu 67 of TBL1 is important for its normal localization.^33^ Therefore, considering that the cleaved sites of TBL1 occur in response to DOX, it is somewhat predictable that there will be aberrant relocalization in the cytosol. Moreover, the intracellular location where cleavage of TBL1 occurs in the DICT condition must also be considered. Taking all of the previous findings together, it is reasonable to infer that the cleavage of TBL1 occurred within the nucleus in response to DOX. However, we have deduced that, following the DOX treatment, the LisH domain, including L67 in TBL1, was lost due to the DICT-triggered cleavage that occurred in the nucleus, and consequently, it was abnormally transferred to the cytosol and was ultimately degraded. Activated caspase-3 is translocated to the nucleus by simple diffusion and disruption of the cytosol-nuclear barrier for its substrates identified in the nucleus in the progression of apoptosis.^41,42^ Indeed, in the DICT condition, TBL1 is abnormally located in the cytosol due to its active caspase-3-mediated cleavage. In general, damaged proteins with aberrant subcellular localization also target ubiquitin-mediated proteasomal degradation to help maintain proteostasis.^43^ In this study, we demonstrated that TBL1^uclv^ is located in the nucleus despite DOX exposure in H9c2 cells, unlike in TBL1^wt^-transfected cells. β-catenin is constantly ubiquitinated in the cytosol under normal conditions, stabilized following Wnt activation, and subsequently translocated to the nucleus.^44^ Therefore, for the interactions between intact TBL1 and β-catenin in DICT, the stabilization and nuclear localization of β-catenin should be investigated following DOX treatment. Notably, we observed for the first time that β-catenin expression was increased and translocated to the nucleus in a dose-dependent manner with DOX. After the DOX treatment, the stabilization and translocation of β-catenin are crucial for its complex formation with TBL1^uclv^. Synthetically, stabilization and normal localization in the nucleus of TBL1 and translocalization of β-catenin under DOX-exposed conditions seem to play a critical role in protecting cardiomyocytes through activating the Wnt/β-catenin signalling pathway.

Finally, we introduced both *ex vivo* (hiPSC-CM) and *in vivo* (mouse) systems to evaluate the effects of intact TBL1 on cardiomyocytes function in the DICT environment. TBL1 cleavage was also observed in hiPSC-CMs following the DOX treatment. In addition, Ad-TBL1^uclv^ overexpression protected hiPSC-CMs from DOX-induced cardiomyocyte death. Monitoring cardiomyocytes’ electrical activity is crucial to investigating cardiac diseases and developing therapeutic strategies.^45^ Our MEA analyses revealed that TBL1^uclv^ overexpression partially ameliorated the disruption of these electrophysiological factors, such as FPD, total active electrodes, beat period, spike amplitude, and conduction velocity following the DOX treatment.^46^ Furthermore, we elucidated that this phenomenon was because intact TBL1, even after DOX exposure, controls the disruption of Ca^2+^ homeostasis, a predominant hallmark related to cardiac functionality.^47^ Current evidence indicates that the diastolic intracellular Ca^2+^ concentration is increased in response to DOX, leading to LV dysfunction.^48^ Our data have shown that the DOX-induced abnormal functional changes in echocardiographic indicators were alleviated in AAV9-TBL1^uclv^-overexpressing mouse hearts via intramyocardial injection. Additionally, DOX-induced fibrosis and apoptotic cell death were significantly lower in AAV9-TBL1^uclv^-overexpressing mouse cardiac tissues than in AAV9-TBL1^wt^-overexpressing tissues.

Irreversible adverse side effects in cancer treatment result in increased patient morbidity and mortality. Cardiotoxicity is the most significant side effect of chemotherapeutic agents. Therefore, ‘prevention’ strategies to avoid it are paramount, and most previous trials have focused on biomarkers.^49^ This study is thus valuable as it expands the selection of preemptive strategies for preventing DICT by presenting a novel target and identifying the molecular mechanisms underlying it. Despite these advantages, our study has several limitations. First, we did not directly compare the phenomenon of TBL1 cleavage in cardiac tissues between normal individuals and patients with DCM. We obtained cardiac tissues from six patients with DCM and two patients with DICT but failed to obtain those from normal individuals. Using hiPSC-CMs as a control group was the best option in this situation. Second, based on only the antibodies recognizing the C-terminal of TBL1 [TBL1(C) and Myc] following DOX treatment, detected endogenous and exogenous cleaved bands of TBL1 appear to have differences. This is inferred because endogenous TBL1, which has a relatively low expression level, was degraded at a faster rate after its cleavage. In addition, differences in cleaved bands of endogenous TBL1 between hiPSC-CMs and H9c2 cells under the same circumstances are presumed to result from species differences. Third, as our study focused on the cross-talk between TBL1 and the Wnt/β-catenin signalling pathway in DICT, direct experimental evidence supporting a correlation between either p53-TBL1 or the p53-Wnt/β-catenin signalling pathway is currently insufficient. Moreover, although two patients supporting our idea were diagnosed with DICT-induced DCMP at 8 and 5 months, after late DOX administration, the two cardiac samples were collected in 2022 and 2017, which is 18 and 5 years after the late DOX dose, respectively, at the time of heart transplantation. Based on the observation from the DICT mouse model utilized in this study, we can predict that the TBL1 cleavage in the samples actually occurred in the early stage of DICT. However, the DICT model using in this study unable to provide clear evidence regarding the impact of the inhibition of it on long-term DICT patients. Therefore, more elaborate and in-depth studies are required. Although this study has limitations, it is clear that our findings have suggested a new molecular mechanism underlying DCM and DICT and related preventive-/therapeutic approaches.

Concluding this investigation, we have identified TBL1 cleavage-induced degradation following DOX exposure and that this process triggers cardiomyocyte death. Specifically, the N-terminus processing of TBL1 by active caspase-3 is crucial for apoptosis induction through the inhibition of Wnt/β-catenin signalling in cardiomyocytes, and it is effectively reversed by the uncleaved mutant form of TBL1 (TBL1^uclv^). These findings were also validated in hiPSC-CMs and *in vivo* DICT mouse models. The results enhance our understanding of TBL1 function and will aid in developing valuable strategies to alleviate DICT during cancer treatment.

**Translational perspective**

The persistence of DICT serves as a major limitation to the clinical application of DOX. Our study underscores the role of TBL1 as a critical regulator of Wnt signalling within the framework of DICT. Interestingly, DOX-mediated inhibition of TBL1 appears to contribute to the onset of cardiac complications. These observations provide valuable insights and identify potential molecular targets for elucidating the underlying mechanisms of DICT arising from clinical Doxorubicin administration.

## Supplementary Material

cvae098_Supplementary_Data

## Data Availability

All original data used for this study are available from the corresponding author upon reasonable request.
