# Supplementary material for: Inhibition of TBL1 cleavage alleviates doxorubicin-induced cardiomyocytes death by regulating the Wnt/β-catenin signal pathway
Source: Cardiovasc Res. 2024 May 9;120(9):1037–50. doi: 10.1093/cvr/cvae098 (PMC11288742; doi:10.1093/cvr/cvae098)
Supplement: cvae098_Supplementary_Data [file cvae098_supplementary_data.docx]

Supplemental Materials for

Inhibition of TBL1 cleavage alleviates doxorubicin-induced cardiomyocytes death by regulating the Wnt/β-catenin signal pathway

Sun-Ho Lee^1^†, Jangho Lee^2^†, Jaewon Oh^3^†, Jin-Taek Hwang^2^, Hae-Jeung Lee^4^, Hwa Kyung Byun^5^, Hyeong-Jin Kim^1^, David Suh^6^, Ho-Geun Yoon^1,7^, Sahng Wook Park^1,7^, Seok-Min Kang^3^, Chulan Kwon^6^, Seung-Hyun Lee^1,6,7^*, Hyo-Kyoung Choi^2^*

Correspondence to: [chkyoung@kfri.re.kr](mailto:chkyoung@kfri.re.kr) (H.-K.C) and [tiger815@yuhs.ac](mailto:tiger815@yuhs.ac) (S.-H.L.)

**This PDF file includes:**

Supplemental Methods

Supplemental Figures S1 to S17

Supplemental Tables S1 to S5

Unedited gel images

Supplemental Methods

In vivo cardiotoxicity model

For a 25 g mouse, DOX was administered by dissolving it fresh in 200 µL of 0.9% saline solution to 0.125 mg/ml for each injection. To induce cardiotoxicity, the prepared 5 mg/kg DOX was administrated intraperitoneally (i.p.) once a week for a duration of 4 weeks (cumulative dose of 20 mg/kg), while saline was injected instead of DOX in the control group. Echocardiography was performed 7 days after the final DOX administration, and tissues were collected for experimentation. Additionally, we calculated the ejection fraction (EF%) and fractional shortening (FS%) based on M-mode echocardiographic images by referring to previous stuides^1^.

## Chromatin immunoprecipitation assays

Briefly, 2 × 10^8^ cells were seeded in 10-mm dishes, treated with 2 μM DOX, and transfected with the indicated constructs. After the first treatment, cells were washed with PBS containing 1% formaldehyde for 10 min and rinsed three times with cold PBS. Cross-linking was stopped by adding 125 mM glycine for 5 min at 24–26 ℃. Subsequent processes were performed following the manufacturer's instructions for the Pierse Agarose ChIP Kit (Thermo Fisher Scientific) using the β-catenin antibody (Cell signaling) but without SDS in any of the buffers. The primers used for amplification are listed in Supplementary Table S4.

## TUNEL assays

For the detection of apoptosis in cells and mouse heart tissues, DNA fragmentation was evaluated using a TdT-mediated dUTP nick end labeling (TUNEL) assay with an *in situ* Cell death Detection Kit, Fluorescein (Sigma-Aldrich), according to the manufacturer's instructions. Briefly, cells were fixed with 4% cold paraformaldehyde in PBS for 30 min, washed with PBS, permeabilized with 0.1% Triton X-100 in 3% BSA for 30 min, and then washed twice with PBS. Cells were labeled with a terminal deoxynucleotidyl transferase enzyme and a label solution (nucleotide mixture in reaction buffer) mixture at 37 ℃ for 60 min in the dark. Subsequently, the rinsed cells with PBS were analyzed under a fluorescence microscope using excitation and detection wavelengths ranging from 450–500 nm and 515–565 nm, respectively.

## Apoptosis assay

After transfection for 48 h, the H9c2 cells were treated with DOX at 37 ℃ for 24 h. The cells were washed twice with PBS and resuspended with Annexin Ⅴ-binding buffers (422201, BioLegend). The cells were then harvested and stained with Annexin Ⅴ-fluorescein isothiocyanate (FITC) (640906, BioLegend) and Zombie NIR^TM^ fixable dye (423105, BioLegend) in the dark in the temperature range of 18 – 22°C for 15 min. Immediately afterward, approximately 10^5^ cells per sample were analyzed using LSR Ⅱ flow cytometer (BD Bioscience) and FLOWJO v10.0.7 software (Tree Star. Inc.)

## Separation of cytoplasmic and nuclear extracts

NE-PER (nuclear and cytoplasmic extraction reagents) (Thermo Fisher Scientific) were used to separate cytoplasmic and nuclear extracts from H9c2 cells, according to the manufacturer's instructions. Briefly, the antibodies for HDAC1 (a nucleus control protein) and tubulin (a cytoplasmic control protein) were used to verify the effectiveness of the separation. The relative levels of the analyzed nuclear proteins were normalized to that of HDAC1, and the relative level of the cytoplasmic proteins was normalized to that of tubulin. The antibodies used for control are listed in Supplemental Table S5.

## TOP/FOP luciferase reporter assays

Approximately 6 × 10^5^ cells were seeded in 60-mm dishes the day before transfection. Cells were then co-transfected with 1 µg of TOPFLASH reporters (Promega) and the relevant expression plasmid containing TBL1. The FOPFLASH luciferase vector (Promega) was used as a control to measure transfection efficiency. TOPFLASH has three Tcf/Lef sites upstream of a promoter and the firefly luciferase gene. FOPFLASH has mutated copies of the Tcf/Lef sites and was used as a control for measuring nonspecific activation of the reporter. To normalize transfection efficiency in the reporter assays, the cells were co-transfected with 0.05 µg of internal control reporter *Renilla reniformis* luciferase driven under the promoter. Luciferase activity was measured according to the manufacturer's instructions.

Plasmid construction, siRNA, and transfection

Various truncated-TBL1 expression plasmids were constructed using standard PCR and then sub-cloned into the pSG5 vector. To generate the Flag and Myc double-tagged TBL1 constructs, the pSG5FlagM2 vector was modified. The primer sequences used for sub-cloning are listed in Table S1. The Flag-TBL1^D125A^-Myc, Flag-TBL1^D136A^-Myc, Flag-TBL1^D215A^-Myc, and Flag-TBL1^uclv^-Myc expression plasmids were derived from Flag-TBL1^wt^-Myc using the QuikChange site-directed mutagenesis kit according to the manufacturer’s instruction (Stratagene, La Jolla, CA, USA). All plasmids were verified by DNA sequencing. Short interfering RNAs (siRNAs) were designed to target caspase-3, caspase-7, and *TBL1* mRNA. Non-targeting siRNA (siControl) was used as a control. Cells were transfected with 200 nM chemically synthesized siRNA (Genolution, Seoul, Korea) using Lipofectamine RNAiMAX reagent (Thermofisher, Waltham, MA, USA) in 60-mm dishes, according to the manufacturer’s instructions. The siRNA sequences used in this study are listed in Supplemental Table S3.

Treatment of cells with inhibitors

Z-VAD (Pan Caspase Inhibitor Z-VAD-FMK; FMK001) and Z-DEVD (Caspase-3 Inhibitor Z-DEVD-FMK; FMK004) were purchased from R&D systems (Minneapolis, MN, USA). Calpain inhibitor-III (MDL-28170; S7394) was purchased from Selleckchem (Houston, TX, USA). The inhibitors were dissolved in DMSO, with Z-VAD and Z-DEVD at a concentration of 50 μM, and Calpain inhibitor-III at 10 μM. These inhibitors, along with DMSO as a control, were added to the medium as indicated. The inhibitors were added to the medium 1 h before DOX treatment. Control experiments were also performed with inhibitors alone for the same duration.

Immunoblot blot, immunoprecipitation, and antibodies

Cells were lysed using Cell Lytic Buffer M (Sigma) supplemented with protease inhibitor (Nachlai Inc, San Diego, CA, USA) and a phosphatase inhibitor cocktail (Calbiochem, Darmstadt, Germany), and 0.1 mM PMSA (Sigma). Total lysates were used for immunoblotting or immunoprecipitation (IP). Antibodies against TBL1 (137083) and HA tags (7392) were purchased from Santa Cruz Biotechnology Inc. (Dallas, TX, USA). MLC2v (10906-1-AP) antibody was purchased from Proteintech Group Inc. (Chicago, IL, USA). Monoclonal ANTI-FLAG M2 (F3165) antibody was obtained from Sigma-Aldrich. β-catenin (#8480), cleaved caspase 3 (#9661), cleaved caspase 7 (#8438), GAPDH (#2118), MYC-tag (#2272), p53 (#9282), and PARP (#9542) antibodies were purchased from Cell Signaling (Beverly, MA, USA). β-actin (47778) antibody was purchased from Abcam (Cambridge, MA, USA). Nkx2.5 (PA5-49431), Titin (27867-I-AP), and Troponin T cardiac (ab10214) antibodies were purchased from Invitrogen (Carlsbad, CA, USA). Detailed information, including the working dilutions of the antibodies used in this study, is summarized in Supplemental Table S5. Immunoblotting images were quantified using ImageJ software (http://rsbweb.nih.gov/ij/). Briefly, to analyze the individual images for quantification, the RGB color images obtained from the immunoblotting analysis were converted to 8-bit grayscale images. The mean gray values and integrated densities were quantified for each object in the images according to the guide instructions provided by ImageJ.

Immunocytochemistry

H9c2 cells were fixed using 4% paraformaldehyde for 15 min, blocked with 5% bovine serum albumin (LPS solution, 9048-46-8, South Korea) with 0.3% Triton X (USB®, 9002-93-1), and incubated overnight at 4°C with primary antibodies for Flag-tag, Myc-tag, and β-catenin. Then, the cells were incubated with Alexa® Fluor 488 chicken anti-rabbit IgG (1:500, Thermo Fisher Scientific, A21441) or Alexa® Flour 546 goat anti-mouse IgG (1:500, Thermo Fisher Scientific, A11030) as the secondary antibody for 3 h at room temperature. DAPI (NucBlue^TM^ Fixed Cell ReadyProbes^TM^ Reagent; Thermo Fisher Scientific, R37606) was used for counterstaining the cell nuclei for 1 min at room temperature. Slides were analyzed with a confocal microscope (LSM710, Zeiss) using ZEN blue edition software. All antibody information is listed in Supplemental Table S5.

*In vitro* translation and caspase cleavage assays

For the *in vitro* caspase-cleavage assays, the indicated plasmids were transcribed and translated using the TNT Quick coupled transcription/translation system (Promega, Madison, WI, USA) in the presence of [_35_S]-methionine (PerkinElmer, Waltham, MA, USA) according to the manufacturer’s instructions. Human recombinant caspase-2, -6, and -9 were purchased from Merk Millipore (Burlington, MA, USA), caspase-3, -8, and -7 were purchased from BD biosciences (Franklin Lakes, NJ, USA), and caspase-10 was purchased from R&D systems (Minneapolis, MN, USA). In 20 µL of caspase reaction buffer [50 mM HEPES, 50 mM NaCl, 0.1 % CHAPS, 10 mM EDTA, 5 % glycerol, 10 mM dithiothreitol (pH 7.2)], the resultant reactant was incubated with 50 ng of each recombinant caspase for 1.5 h at 37 ℃. After adding 5 µL of the 5 × sample buffer [250 mM Tris-HCl, 500 mM dithiothreitol, 10% SDS, 0.5% bromphenol blue, 50% glycerol (pH 6.8)], the reactions were halted by boiling for 5 min. The proteins were then isolated using SDS-PAGE and autoradiography.

Duolink *in situ* proximity ligation assay analysis

Duolink in situ PLA analysis was performed according to the manufacturer’s instructions (Sigma-Aldrich, DUO92101). Briefly, 4% cold paraformaldehyde-fixed cells were washed with PBS, incubated for 15 min in PBS supplemented with 0.3% Triton X-100, washed, and then blocked with blocking solution. Primary antibodies were added, and the cells were incubated with PLUS and MINUS secondary PLA probes against either rabbit or mouse IgGs. Following incubation, hybridization, ligation, and amplification steps were successively performed. Samples were then mounted and examined using a Zeiss LSM710 confocal microscope (Carl Zeiss, Oberkochen, Germany).

RNA isolation and quantitative RT-PCR

Total RNA was extracted from cells using a Ribospin™ total RNA purification kit (GeneAll Biotechnology, Seoul, Korea). Reverse transcription was performed with PrimeScript^TM^ Reverse Transcriptase (Takara, San Jose, CA, USA), according to the manufacturer's instructions. Genes were amplified on a QuantStudioTM 3 Real-Time PCR system (Applied Biosystems^TM^, Waltham, MA, USA) using FastStart Universal SYBR® Green Master (Roche, Basel, Swiss).

Adenovirus

For stable overexpression of either TBL1^wt^ or TBL1^uclv^ in iPSC-CMs, the TBL1 was constructed using a standard PCR protocol and sub-cloned into the pEntCMV-EF1a-GFP shuttle vector. The mouse TBL1^uclv^ plasmid (Ad-TBL1^uclv^) was generated using site-directed mutagenesis with pEntCMV-TBL1^wt^-EF1a-GFP (Ad-TBL1^wt^) as the template. The plasmids were then verified by DNA sequencing. Virus production was outsourced to Koma Biotechnology (Seoul, Korea). Briefly, viruses were grown on a monolayer of HEK 293 cells. When the inoculated cells exhibited pronounced cytopathic effects, virus-containing media was centrifuged at 1500 rpm for 10 min. Viruses were then purified on two sequential cesium chloride gradients and dialyzed with PBS containing 10% glycerol (pH 7.4) to reduce the salt concentration. The titers of the purified viruses were measured using A_260_. Ten microliters of the viral sample were then added to 990 µL of 0.1% SDS buffer and incubated in the temperature range of 18 – 22°C for 15 min, and the OD value was measured at A_260_. The adenovirus titer was calculated as follows: Adenovirus titer in viral particles (vp)/mL = OD value × dilution factor × 1.1 × 10^12^.

Adeno-associated virus (AAV)

For the stable overexpression of either TBL1^wt^ or TBL1^uclv^ in mice hearts, the TBL1 was constructed using a standard PCR protocol and sub-cloned into the Virovek’s AAV shuttle vector (Hayward, USA). The mouse TBL1^uclv^ plasmid (AAV9-TBL1^uclv^) was generated using site-directed mutagenesis with pFB-CMV-TBL1^wt^-T2A-GFP-WRE-bGHpA (AAV9-TBL1^wt^) as the template. The plasmids were then verified by DNA sequencing. Virus production was outsourced to Koma Biotechnology (Seoul, Korea). Briefly, Sf9 insect cells were cultured in Sf-900 II serum-free medium. Triple infection with baculovirus vector coding for the replication proteins, the structural proteins, and AAV vector genomes was conducted to produce AAV9 and AAV-PHP.B vectors. After 3 days of infection, the cells were centrifuged at 3000 rpm for 15 min. The lysates were then loaded onto a SW28 centrifuge tube containing sequential cesium chloride gradients for centrifugation at 100,000 g for 16 h. The rAAV was dialyzed with PBS with a PD-10 desalting column (GE HealthCare, Piscataway). The titers of the purified viruses were measured using A_260_. Ten microliters of the viral sample was added to 990 µL of 0.1% SDS buffer and incubated in the temperature range of 18 – 22°C for 15 min, and the OD value was measured at A_260_. The adeno-associated virus titer was calculated as follows:

Adeno-associated virus titer in viral particles (vp)/mL = OD value × dilution factor × 1.1 × 10^12^.

Measurement of intracellular calcium

iPSC-derived cardiomyocytes were seeded in matrigel-coated 35-mm glass bottom dishes (MatTek, P35G-1.5-20-C). Cardiomyocytes were loaded with 5 μM Rhod-2 AM fluorescent calcium indicator (Thermo Fisher Scientific, R1244) for 20 min and then recorded in Tyrode’s solution (Sigma, T2397) in the temperature range of 18 – 22°C. Spontaneous Ca^2+^ transients were acquired using a line scan mode. A total of 10,000 lines were acquired over a 61 s recording. For the Ca^2+^ transient recording, confocal line-scan imaging was performed on resting cells at 552 nm excitation and 581 nm emission using a Zeiss 710 inverted confocal microscope (Carl Zeiss) at 20× magnification. The Ca^2+^ transients were processed using ZEN software and analyzed using Microsoft Excel.

Immunohistochemistry, interpretation, and scoring

Cardiac tissues from six patients who died of DCM were used for IHC. Detailed patient information is provided in Table S4. The tissues were fixed in 4% formalin, embedded in paraffin, and cut into 4–5 µm-thick sections. Immunohistochemistry was then performed using a Klear Mouse HRP with a DAB kit (GBI Lab, Bothell, WA, USA) according to the manufacturer’s protocol. Briefly, for antigen retrieval, slides were heated in citrate butter (pH 6.0) for 30 min and exposed to Protein Block Serum-Free blocking solution (Dako, Carpinteria, CA, USA) to minimize non-specific background staining. The primary antibody, anti-TBL1 (N-term) (1:100), was incubated overnight at 4 ℃ and visualized using 3,3'-diaminobenzidine (DAB). The stained DAB intensities were quantified using the IHC profiler plugin in ImageJ software.

Hematoxylin and eosin, and Masson’s trichrome staining

The cardiac specimens were fixed using 4% buffered paraformaldehyde, embedded in paraffin, and cut into 4–5-µm-thick sections, which were stained with hematoxylin and eosin (H&E). Masson’s trichrome staining (MTS) was performed using an MT kit (StatLab, American MasterTech, Lodi, CA, USA) according to the manufacturer’s instructions. Briefly, the embedded sections were deparaffinized and rinsed with 100% ethanol and water, respectively. The slides were incubated with Bouin’s fluid at 4 ℃ for 1 h. Subsequently, they were sequentially incubated with Weiger’s working hematoxylin, Biebrich scarlet acid fuchsin, phosphomolybdic/phosphotungstic acid, aniline blue stain, and 1% acetic acid. Finally, the slides were dehydrated and mounted. Fibrosis in the cardiac tissues was assessed using fluorescent microscopy (OLYMPUS, IX71).

Supplemental Figures


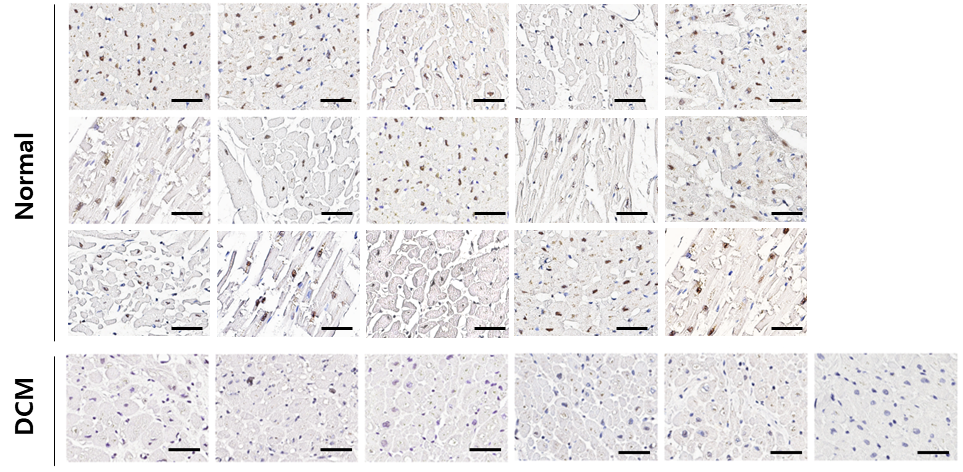


Supplemental Figure S1.

**TBL1 expression in normal individuals and patients with dilated cardiomyopathy.** A total of 15 normal cardiac tissues and 6 dilated cardiomyopathy (DCM) cardiac tissues were stained with anti-TBL1 using immunohistochemistry (IHC). IHC revealed that TBL1 expression was lower in the DCM group than in normal groups. Scale bar = 50 µm.


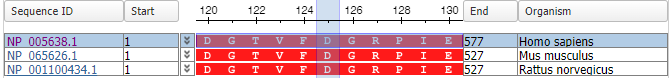

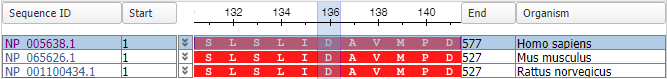

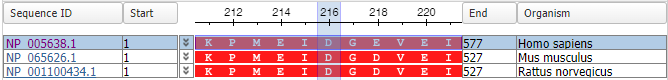


*NCBI, Multiple Sequence Alignment Viewer 1.24.1*

Supplemental Figure S2.

**The sequence alignment of TBL1 cleavage sites.** Sequence alignment of the TBL1 cleavage sites in human, mouse, and rat was conducted using Multiple Sequence Alignment tool (COBALT, <https://www.ncbi.nlm.nih.gov/tools/cobalt/re_cobalt.cgi>), and the result was visualized with Multiple Sequence Alignment viewer 1.24.

Supplemental Figure S3.

**Computational prediction of cleavage sites in the conserved sequences of TBL1.** The cleavage probability of TBL1 was predicted using CaspDB^2^ (<http://caspdb.sanfordburnham.org>). P4-P2’ indicates target sequences in TBL1. Cleavage was predicted to occur between P1 and P1′. The seven predicted cleavage target sequences are highlighted in red (upper panel). Specific information, including specificity for the seven predicted positions, is listed in the table (lower panel).


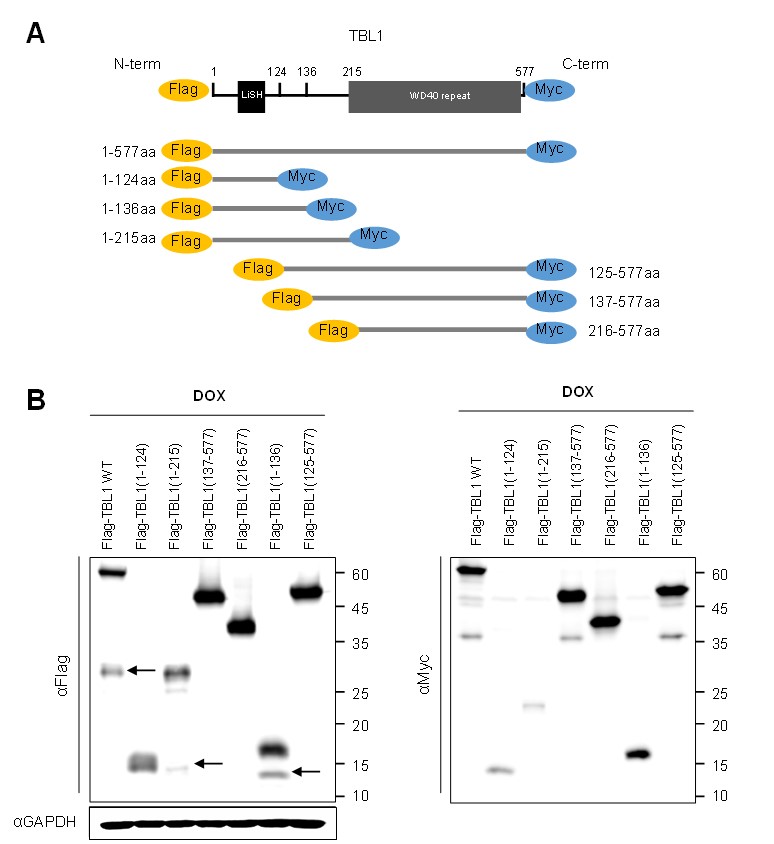


Supplemental Figure S4.

**TBL1 plasmid construction strategy. A.** Various plasmids of Flag- and Myc-tagged truncated TBL1 variants were constructed to identify cleavage sites in TBL1. B**.** DOX-induced TBL1 cleavage occurred within 1–215 amino acid residues. The indicated constructs of Flag-truncated TBL1-Myc, including Flag-TBL1^wt^-Myc, were transiently transfected into H9c2 cells. The cells were incubated with 2 µM DOX for 24 h. Cells were harvested, total protein was extracted, and then immunoblotted with the Flag or Myc antibody. The arrows indicate cleaved TBL1.

Supplemental Figure S5.

**Schematic representation of the expected cleaved TBL1 forms.** The diagrams show the possible cleavage of the TBL1^wt^ construct. For the TBL1^D125A^, TBL1^D136A^, and TBL1^D215A^ mutants, cleavage is expected at the remaining two positions except for the mutation site.

Supplemental Figure S6.

**Schematic representation showing TBL1 detection using the Duolink *in situ* proximity ligation assay (PLA) reaction.** PLA is a technique that directly detects protein–protein interactions *in situ* at distances < 40 nm^3^. The working principle of PLA was applied to observe the cleavage of TBL1. The two primary antibodies against Flag and Myc from different species were used to recognize specific protein targets, tagged at the N- and C-terminus of TBL1, respectively (6-1). PLA probes consist of a pair of oligonucleotide-labeled secondary antibodies that bind to the primary antibodies (6-2). Hybridizing connector oligos join the PLA probes only if Flag and Myc are in close proximity to each other at the molecular level (6-3). Ligase forms a closed, circle DNA template required for RCA (6-4). Finally, fluorochrome tagged-complementary detection oligos are hybridized to repeat sequences in the amplicons, and consequently, red dots develop (6-5).


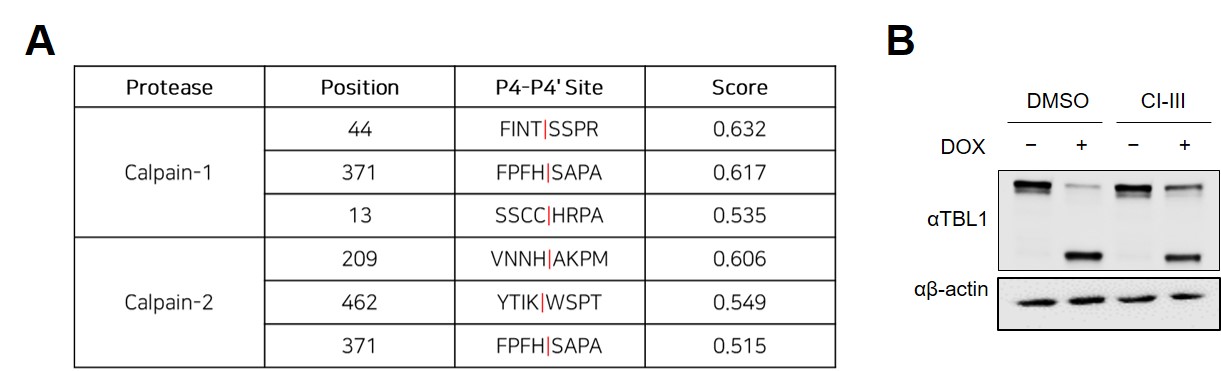


Supplemental Figure S7.

**Computational prediction of cleavage sites in the conserved sequences of TBL1.** A. The cleavage probability of TBL1 was predicted using Procleave^4^ (procleave.erc.monash.edu/). “P4-P4' Site” indicates the target sequences in TBL1. Cleavage was predicted to occur between P1 and P1'. The seven predicted cleavage target sites are highlighted in red. B. DOX-induced TBL1 cleavage is independent of calpain. H9c2 cells were exposed to 10 µM of Calpain inhibitor-III, which inhibits both calpain-1 and calpain-2, with or without 2 µM of DOX for 24 h. The cells were lysed, and protein extracts were used for immunoblot assays with the indicated antibodies.


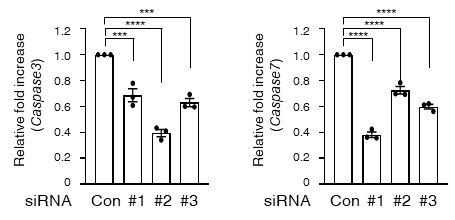


Supplemental Figure S8.

**Validation of caspase-3 and caspase-7 siRNAs.** For siRNA validation, the indicated siRNAs were transfected (at a concentration of 10 pmol) into H9c2 cells. After 48 h, total RNA was extracted, cDNA was synthesized, and the qRT-PCR was performed. The most efficient siRNA was used in the experiment.

Supplemental Figure S9.

***In vitro* translated-TBL1 is cleaved by either caspase-3 or caspase-7 in a cell-free system.** TBL1 was labeled with ^35^S-labeled methionine (^35^S-Met) through *in vitro* translation and subsequently used as a substrate for caspase detection assays. ^35^S-labeled TBL1 was detected by autoradiography. The arrows indicate cleaved TBL1 bands.

Supplemental Figure S10.

**Predicted D125, D136, and D215 positions in TBL1 that are responsible for caspase-3-dependent cleavage.** TBL1^uclv^ mutant is not cleaved by caspase-3 in a cell-free system. TBL1^wt^, TBL1^D125A^, TBL1^D136A^, TBL1^D215A^, and TBL1^uclv^ mutants were labeled with ^35^S using *in vitro* translation and sequentially incubated with the recombinant caspase-3 enzyme. ^35^S-labeled TBL1 was detected by autoradiography. The arrows indicate cleaved-TBL1 bands.

Supplemental Figure S11.

**Activation of the Wnt/β-catenin signaling pathway inhibits DOX-induced apoptotic cell death.** H9c2 cells were exposed to LiCl (2.5 mM) or CHIR99021 (3 uM), which are Wnt agonists, in the presence of DOX for 24 h. Representative image of TUNEL-positive apoptotic cells (red) versus DAPI (blue). Scale bar = 20 µm. The histogram shows the percentage of TUNEL-positive cells relative to DAPI-positive cells, representing the quantification of apoptosis. The values are presented as the mean ± S.D. from three independent experiments. **** *p* < 0.0001 (Student’s *t*-test).

Supplemental Figure S12.

**TBL1 mutants did not activate Wnt/β-catenin signaling.** A. TBL1 mutants were not able to enhance Wnt reporter gene activity. The indicated TBL1 constructs with TOP/FOP FLASH reporters were co-transfected into H9c2 cells for 48 h under LiCl-exposed conditions. Whole-cell lysates were used for luciferase assays. The results are represented as the mean ± S.D. from three independent experiments. * *p* < 0.05, ** *p* < 0.01, *** *p* < 0.001, and **** *p* < 0.0001 (Student’s *t*-test). B. TBL1 mutants failed to increase in *Axin2* mRNA expression. The indicated TBL1 constructs were transfected into H9c2 cells for 48 h under LiCl-exposed conditions. cDNA was synthesized from mRNA, and the levels of *Axin2* were analyzed using real-time PCR. **** *p* < 0.0001 (Student’s *t*-test).


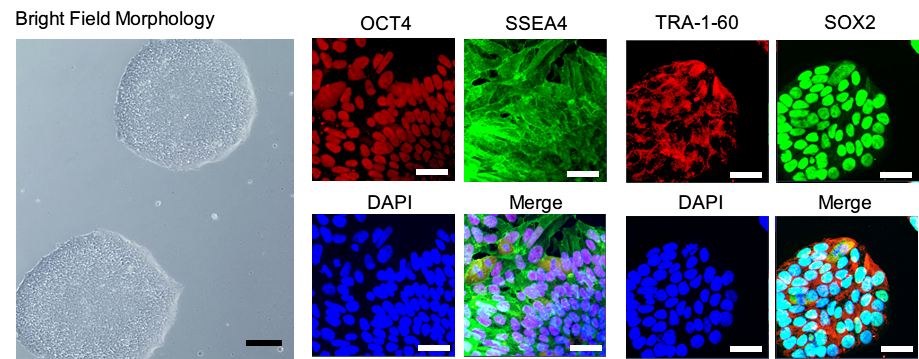


Supplemental Figure S13.

**Validation of human induced pluripotent stem cells (hiPSCs). a.** To validate hiPSC, hiPSC markers, SSEA4, OCT4, TRA-1-60, and SOX2, were immunostained as described in the Materials and Methods section. Representative images of three independent experiments are shown. Scale bar = 50 µm.


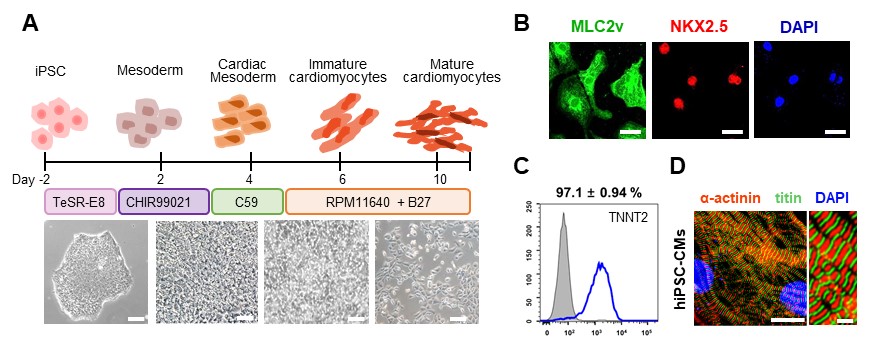


Supplemental Figure S14.

**Validation of the human induced pluripotent stem cell-derived cardiomyocytes (hiPSC-CMs).** (A) Schematic representation of the hiPSC-CMs differentiation. The validated hiPSCs were cultured in the indicated media during the differentiation period and finally differentiated to matured hiPSC-CMs through cardiac mesoderm, cardiac progenitors, and immature CMs. Changes in cell morphology according to differentiation are demonstrated. (B) To validate hiPSC-CMs, hiPSC-CMs were stained with the indicated antibodies recognizing hiPSC-CM markers, and immunofluorescence was analyzed as described in the Materials and Methods (upper right panel). Scale bar = 50 µm. (C) TNNT2-positive cells were assessed using flow cytometry (lower right panel). (D) To confirm hiPSC-CM, hiPSC-CMs were stained with the MLC2V (ventricular) and TNNT2 antibodies, and immunofluorescence analysis was performed. Scale bar = 50 µm**.**

Supplemental Figure S15.

**Ad-TBL1^wt^ and -TBL1^uclv^ expression identification efficiency.** Ad-Flag-TBL1^wt^-GFP or -Flag-TBL^uclv^-GFP (1×10^12^ vp/mL, respectively) was inoculated in HL-1 mouse cardiac muscle cells with a multiplicity of infection (MOI) of 100. After 72 h, the infected cells were observed under a fluorescence microscope (upper panel). Cells were harvested and analyzed to extract protein, and immunoblot assays were performed with the indicated antibodies (lower panel).

Supplemental Figure S16.

**Experimental design.** Representative image of hiPSC-CMs cultured on a microelectrode array (MEA).

Supplemental Figure S17.

**AAV9-TBL1^wt^ and -TBL1^uclv^ expression identification efficiency.** AAV9-Flag-TBL1^wt^-GFP or -Flag-TBL^uclv^-GFP (1×10^12^ vp/mL, respectively) was inoculated in HL-1 mouse cardiac muscle cells with a multiplicity of infection (MOI) of 100. After 72 h, the infected cells were observed under a fluorescence microscope (upper panel). Cells were harvested and analyzed to extract protein, and immunoblot assays were performed with the indicated antibodies (lower panel).

Supplemental Tables

Supplemental Table S1.

Clinical information for patients with dilated cardiomyopathy

|  | **Patient** | | **Tissue** | | | |
| --- | --- | --- | --- | --- | --- | --- |
|  | **Gender** | **Age** | **Diagnosis** | **Location** | **LVEF (%)** | **LVEDD (mm)** |
| Normal | Male | 33 | Normal | - |  |  |
|  | Male | 24 | Normal | - |  |  |
|  | Male | 40 | Normal | - |  |  |
|  | Female | 54 | Normal | - |  |  |
|  | Male | 50 | Normal | - |  |  |
|  | Male | 40 | Normal | - |  |  |
|  | Female | 21 | Normal | - |  |  |
|  | Female | 21 | Normal | - |  |  |
|  | Male | 30 | Normal | - |  |  |
|  | Male | 40 | Normal | - |  |  |
|  | Male | 47 | Normal | - |  |  |
|  | Male | 45 | Normal | - |  |  |
|  | Male | 38 | Normal | - |  |  |
|  | Male | 45 | Normal | - |  |  |
|  | Male | 16 | Normal | - |  |  |
| DCM | Male | 41 | idiopathic DCMP | LV | 24 | 60 |
|  | Male | 49 | idiopathic DCMP | LV | 17 | 78 |
|  | Male | 65 | idiopathic DCMP | LV | 28 | 66 |
|  | Male | 63 | idiopathic DCMP | LV | 18 | 58 |
|  | Male | 66 | idiopathic DCMP | LV | 14 | 79 |
|  | Male | 57 | idiopathic DCMP | LV | 16 | 64 |

Supplemental Table S2.

|  | **Patients** | | **Dx.** | **CTx**  **regimen** | **Duration of**  **CTx** | **Dosage of Doxorubicin** | **Date of DCMP Dx.**  **(LVEF)** | **Date of**  **heart transplantation** |
| --- | --- | --- | --- | --- | --- | --- | --- | --- |
|  | **Gender** | **Age*** |  |  |  |  |  |  |
| DICT 1 | Female | 61 | Non-Hodgkin lymphoma | CHOP (cyclophosphamide, vincristine, doxorubicin, and Prednisolone) | Mar 2004 – Oct 2004 | 50 mg/m^2^/dose * 6  Total cumulative dose: 300 mg/m^2^ | Feb 2005  (LVEF 22%) | Apr 2022 |
| DICT 2 | Female | 66 | Endometrial cancer | Doxorubicin+carboplatin | Dec 2011 – Apr 2012 | 60 mg/m^2^/dose * 6  Total cumulative dose: 360 mg/m^2^ | June 2012  (LVEF 29%) | July 2017 |

Abbreviations: DICT, Doxorubicin-induced cardiotoxicity; CTx, Chemotherapy treatment; DCMP, Dilated cardiomyopathy; Dx., Diagnosis; LVEF, Left ventricular ejection fraction
* The age at the time of heart transplantation.

Supplemental Table S3.

siRNA sequences used in this study

| **Name** |  | **Sequences** |
| --- | --- | --- |
| siControl | Forward | 5’ CCUCGUGCCGUUCCAUCAGGUAGUU 3’ |
|  | Reverse | 5’ CUACCUGAUGGAACGGCACGAGGUU 3’ |
| siCasp3 #1 | Forward | 5’ GAGAUGGGUUUAUGUAUAAUU 3’ |
|  | Reverse | 5’ UUAUACAUAAACCCAUCUCAG 3’ |
| **siCasp3 #2** | Forward | 5’ GCCGACUUCCUGUAUGCUUAC 3’ |
|  | Reverse | 5’ GUAAGCAUACAGGAAGUCGGC 3’ |
| siCasp3 #3 | Forward | 5’ GGCCUGCCGUGGUACAGAAUU 3’ |
|  | Reverse | 5’ UUCUGUACCACGGCAGGCCUG 3’ |
| **siCasp7 #1** | Forward | 5’ GAUCCUGACCAGGGUGAAC 3’ |
|  | Reverse | 5’ GUUCACCCUGGUCAGGAUC 3’ |
| siCasp7 #2 | Forward | 5’ GGGCAAAUGCAUCAUAAUAUU 3’ |
|  | Reverse | 5’ UAUUAUGAUGCAUUUGCCCAG 3’ |
| siCasp7 #3 | Forward | 5’ GGAACUCUACUUCAGUCAAUU 3’ |
|  | Reverse | 5’ UUGACUGAAGUAGAGUUCCUU 3’ |
| siTBL1 | Forward | 5’ GUAGACAAGACAACAAUAAUU 3’ |
|  | Reverse | 5’ UUAUUGUUGUCUUGUCUACUU 3’ |

Abbreviations: Casp3, caspase-3; Casp7, caspase-7; TBL1, transducin beta like 1

Supplemental Table S4.

Primers used for qRT-PCR and ChIP assays.

| **Name** |  | **Sequences** |
| --- | --- | --- |
| *Axin2* | Forward | 5’ TAGGCGGAATGAAGATGGGC 3’ |
|  | Reverse | 5’ GTCCGGAAGAGGTATGCACC 3’ |
| *c-myc* | Forward | 5’ CCAGCAGCGACTCTGAAGAAG 3’ |
|  | Reverse | 5’ GATGACCCTGACTCGGACCTC 3’ |
| *Axin2* (ChIP) | Forward | 5’ CTGGAGCCGGCTGCGCTTTGATAA 3’ |
|  | Reverse | 5’ CGGCCCCGAAATCCATCGCTCTGA 3’ |
| *c-myc* (ChIP) | Forward | 5’ AAGATCCTCTCTCGCTAATCTCC 3’ |
|  | Reverse | 5’ AGAAGCCCTGCCCTTCTC 3’ |
| *GAPDH* | Forward | 5’ TGATCTACCCACGGCAAGTT 3’ |
|  | Reverse | 5’ TGATGGGTTTCCCGTTGATGA 3’ |

Abbreviations: ChIP, chromatin immunoprecipitation; GAPDH, glyceraldehyde-3-phosphate dehydrogenase

Supplemental Table S5.

Antibodies used in this study.

| **Target** | **Supplier** | **Cat no.** | **Dilution** | **Usage** |
| --- | --- | --- | --- | --- |
| TBL1  (n_119-148) | Santa Cruz | 365661 | 1:1000 | WB/IP  /ChIP |
| TBL1  (c_211-577) | Santa Cruz | 137083 | 1:1000 |  |
| Flag | Sigma Aldrich | A8592 | 1:10000 |  |
| Myc | Cell Signaling Technology | 2278 | 1:1000 |  |
| HA | Santa Cruz | 7392 | 1:1000 |  |
| β-Actin | Santa Cruz | 47778 | 1:1000 |  |
| Casp-3 (clv) | Cell Signaling Technology | 9661 | 1:1000 |  |
| Casp-7 (clv) | Cell Signaling Technology | 8438 | 1:500 |  |
| clv.PARP-1 | Cell Signaling Technology | 9542 | 1:1000 |  |
| p53 | Cell Signaling Technology | 2524 | 1:1000 |  |
| β-catenin | Cell Signaling Technology | 8480 | 1:1000 |  |
| HDAC1 | Cell Signaling Technology | 5356 | 1:1000 |  |
| Tubulin | Cell Signaling Technology | 3873 | 1:1000 |  |
| TBL1 | Santa Cruz | 365661 | 1:100 | IF |
| Flag | Sigma Aldrich | F3165 | 1:100 |  |
| Myc | Cell Signaling Technology | 2278 | 1:1000 |  |
| β-catenin | Cell Signaling Technology | 8480 | 1:100 |  |
| SSEA4 | Thermo Fisher Scientific | 46-8843-42 | 1:100 |  |
| OCT4 | Cell Signaling Technology | 9656 | 1:300 |  |
| TRA-1-60 | Thermo Fisher Scientific | 13-8863-82 | 1:100 |  |
| SOX2 | Thermo Fisher Scientific | 53-9811-82 | 1:100 |  |
| NKX2.5 | Thermo Fisher Scientific | PA5-49431 | 1:100 |  |
| Titin | Proteintech | 27867-1-AP | 1:100 |  |
| α-actinin | Thermo Fisher Scientific | A7811 | 1:100 |  |
| MLC2V | Proteintech | 10906-1-AP | 1:500 |  |

Abbreviations: TBL1, transducin beta like 1; n, N-terminus; c, C-terminus; HA, haemagglutinin; Casp3, caspase-3; Casp7, caspase-7; clv, cleavage; PARP1, poly(ADP-ribose) polymerase 1; p53, tumor protein P53; HDAC1, histone deacetylase 1; SSEA4, stage-specific embryonic antigen 4; OCT4, octamer-binding transcription factor 4; TRA-1-60, podocalyxin; Sox2, SRY-box transcription factor 2; NKX2.5, NK2 homeobox 5; MLC2v, myosin light chain 2, ventricular/cardiac muscle isoform; WB, Western blot assay; IP, immunoprecipitation; ChIP, chromatin immunoprecipitation; IF, immunofluorescence assay

# References

1. Gao S, Ho D, Vatner DE, Vatner SF. Echocardiography in Mice. *Curr Protoc Mouse Biol* 2011;**1**:71-83.

2. Kumar S, van Raam BJ, Salvesen GS, Cieplak P. Caspase cleavage sites in the human proteome: CaspDB, a database of predicted substrates. *PLoS One* 2014;**9**:e110539.

3. Alam MS. Proximity Ligation Assay (PLA). *Curr Protoc Immunol* 2018;**123**:e58.

4. Li F, Leier A, Liu Q, Wang Y, Xiang D, Akutsu T, Webb GI, Smith AI, Marquez-Lago T, Li J, Song J. Procleave: Predicting Protease-specific Substrate Cleavage Sites by Combining Sequence and Structural Information. *Genomics Proteomics Bioinformatics* 2020;**18**:52-64.

**Unedited gel images**

**
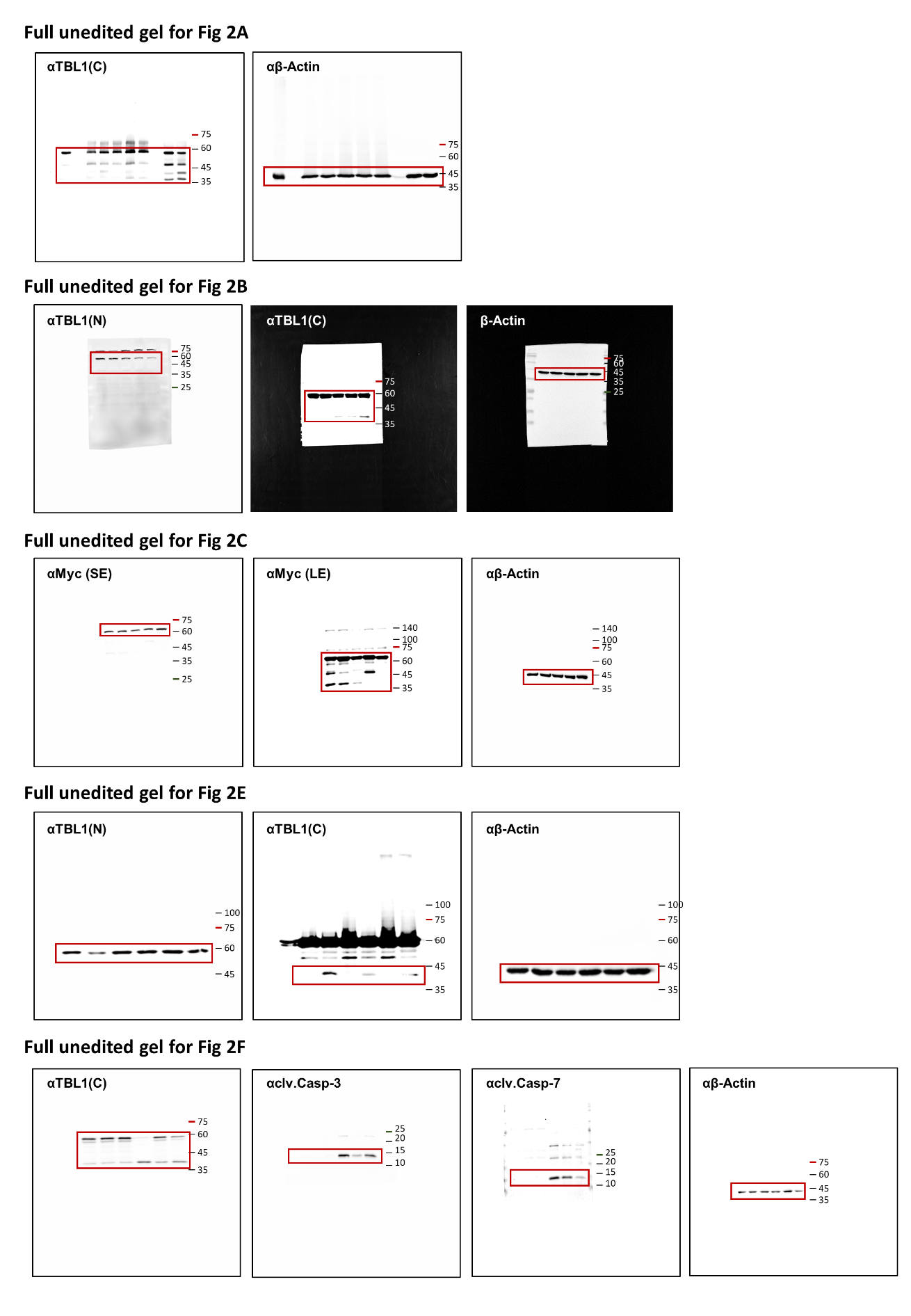
**

**
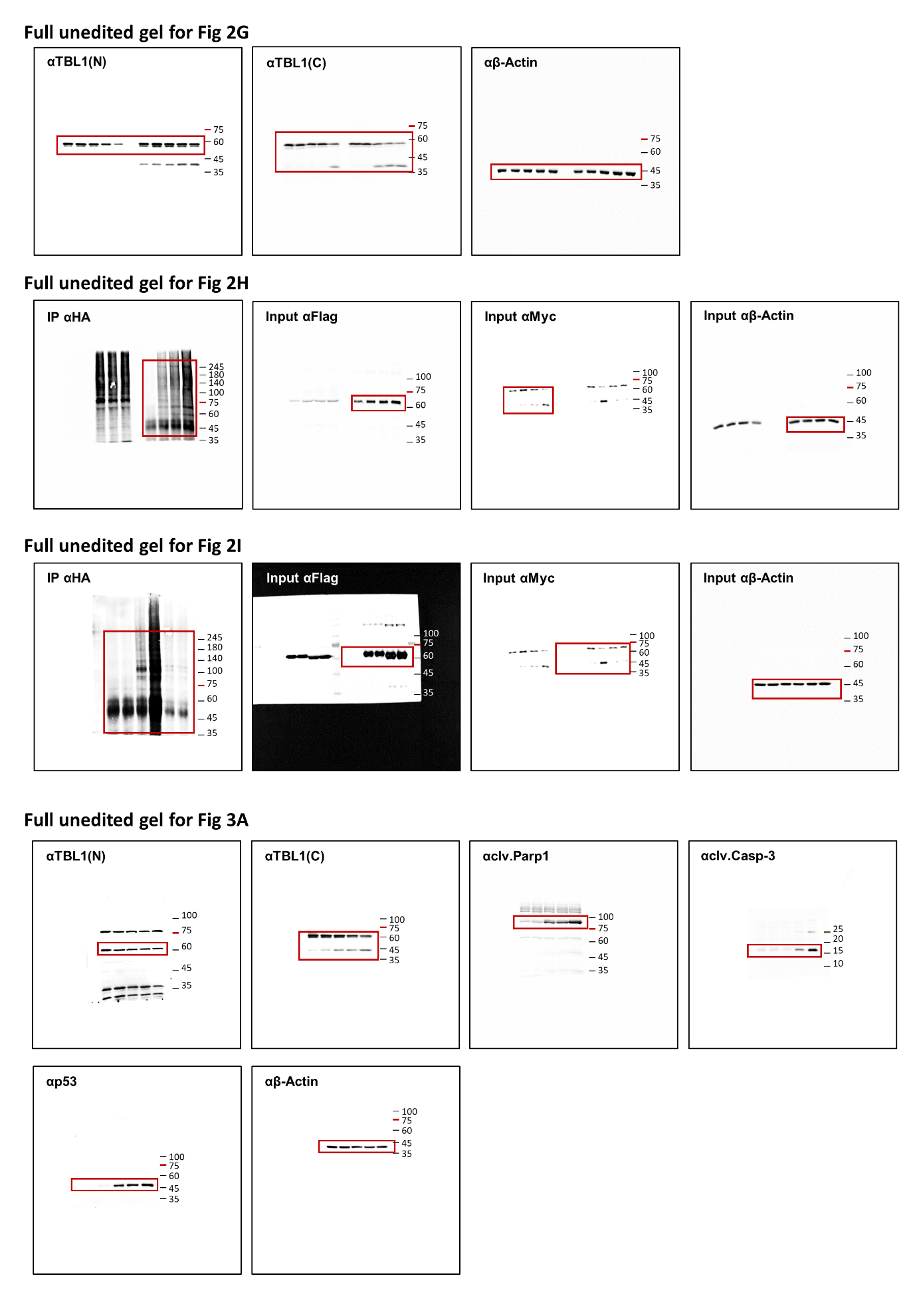
**

**
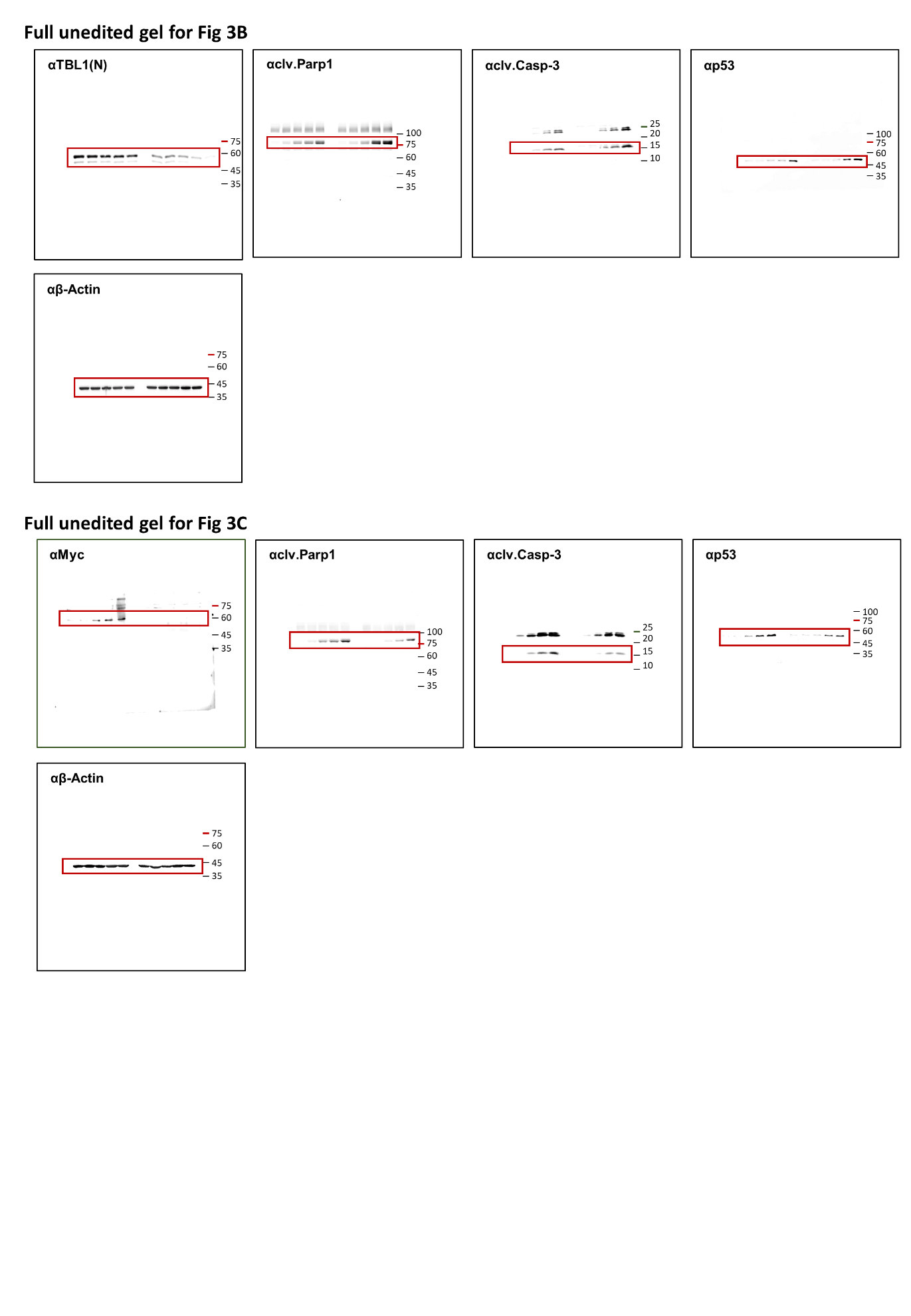
**

**
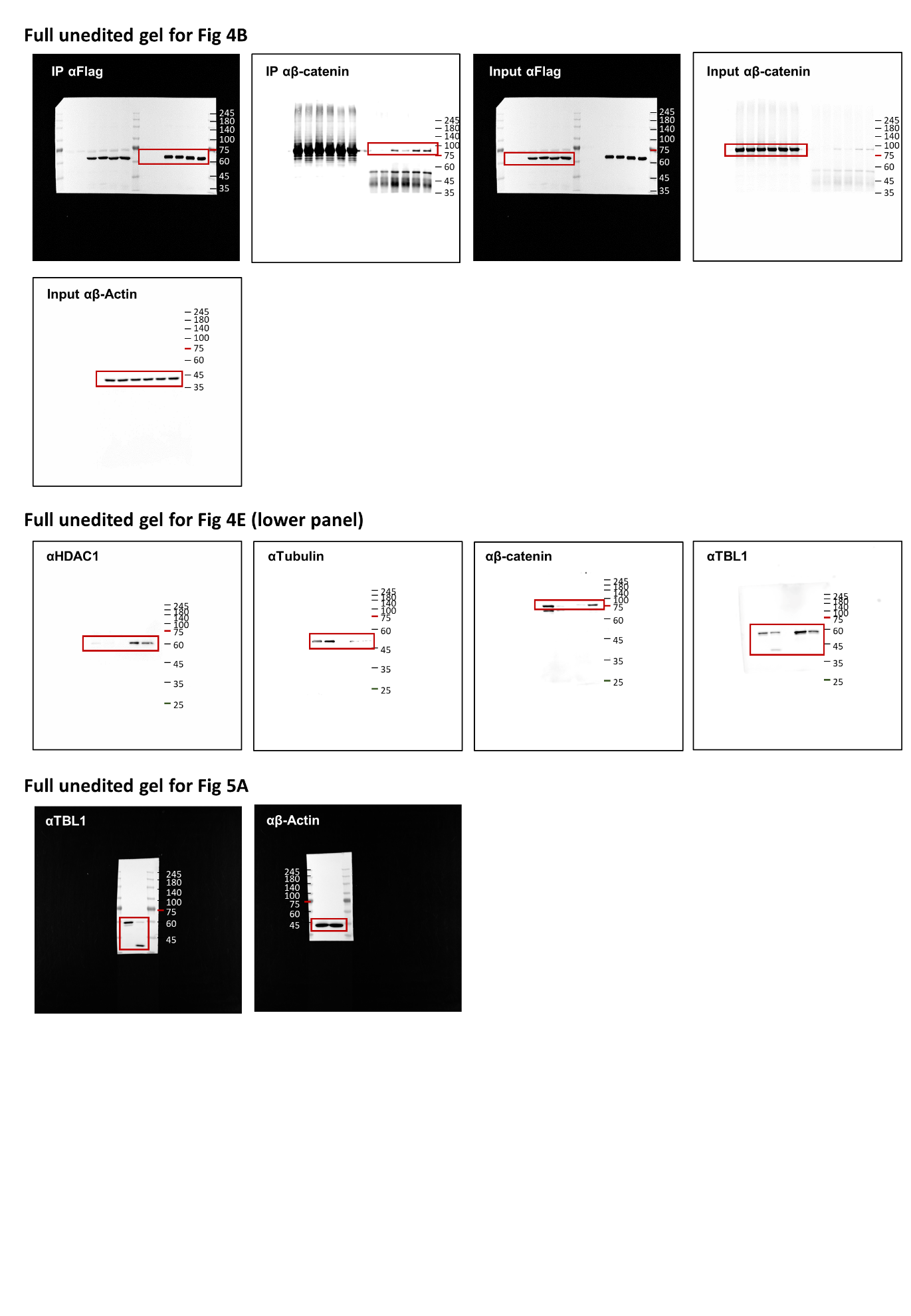
**

**
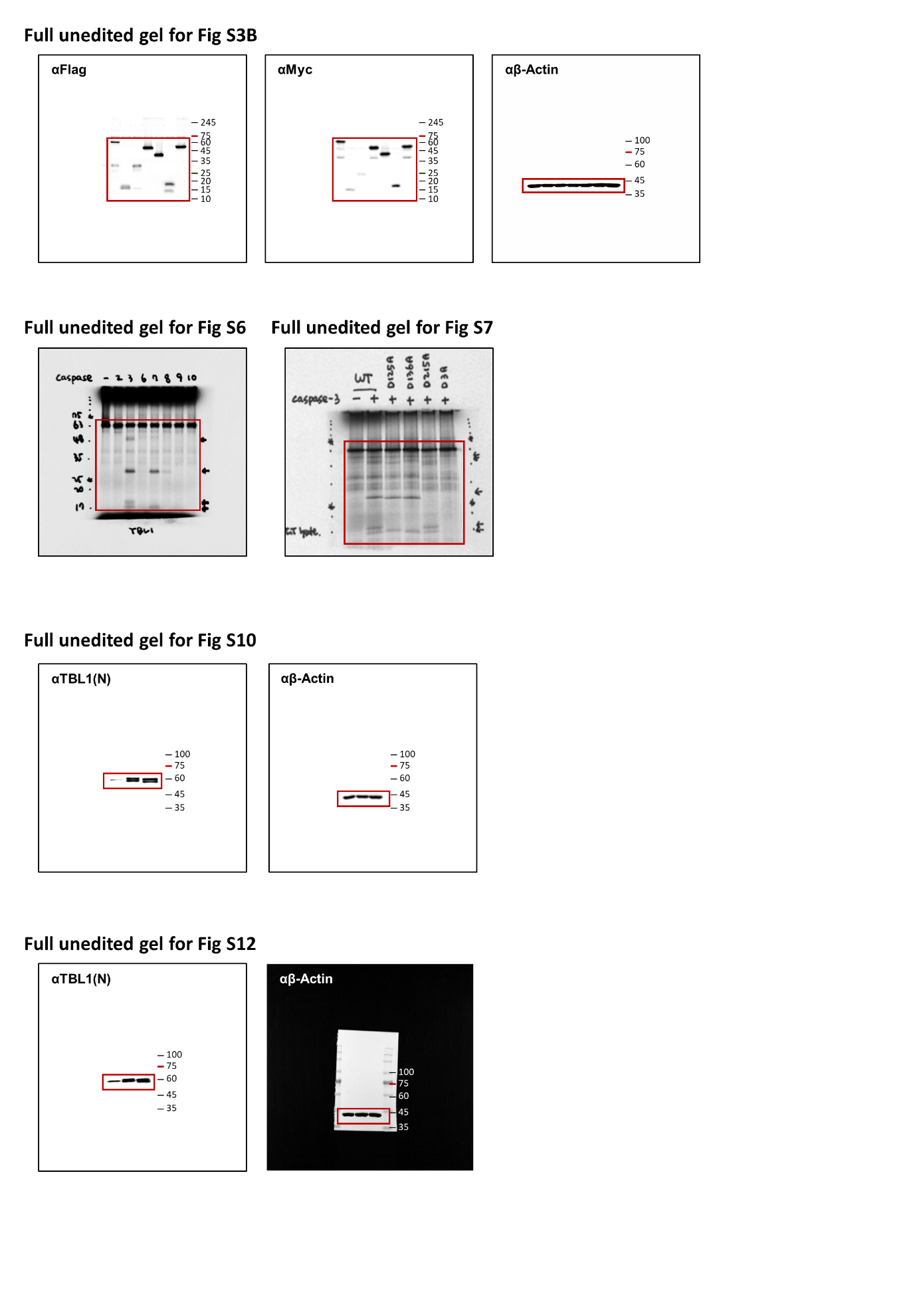
**
